# Spiking in auditory cortex following thalamic stimulation is dominated by cortical network activity

**DOI:** 10.3389/fnsys.2014.00170

**Published:** 2014-09-19

**Authors:** Bryan M. Krause, Aeyal Raz, Daniel J. Uhlrich, Philip H. Smith, Matthew I. Banks

**Affiliations:** ^1^Neuroscience Training Program, University of WisconsinMadison, WI, USA; ^2^Department of Anesthesiology, School of Medicine and Public Health, University of WisconsinMadison, WI, USA; ^3^Department of Anesthesiology, Rabin Medical Center, Petah-Tikva, Israel, affiliated with Sackler School of Medicine, Tel Aviv UniversityTel Aviv, Israel; ^4^Department of Neuroscience, School of Medicine and Public Health, University of WisconsinMadison, WI, USA

**Keywords:** auditory cortex, thalamo-cortical (TC), UP states, canonical microcircuit, calcium imaging, multiunit activity, patch clamp

## Abstract

The state of the sensory cortical network can have a profound impact on neural responses and perception. In rodent auditory cortex, sensory responses are reported to occur in the context of network events, similar to brief UP states, that produce “packets” of spikes and are associated with synchronized synaptic input (Bathellier et al., 2012; Hromadka et al., 2013; Luczak et al., 2013). However, traditional models based on data from visual and somatosensory cortex predict that ascending sensory thalamocortical (TC) pathways sequentially activate cells in layers 4 (L4), L2/3, and L5. The relationship between these two spatio-temporal activity patterns is unclear. Here, we used calcium imaging and electrophysiological recordings in murine auditory TC brain slices to investigate the laminar response pattern to stimulation of TC afferents. We show that although monosynaptically driven spiking in response to TC afferents occurs, the vast majority of spikes fired following TC stimulation occurs during brief UP states and outside the context of the L4>L2/3>L5 activation sequence. Specifically, monosynaptic subthreshold TC responses with similar latencies were observed throughout layers 2–6, presumably via synapses onto dendritic processes located in L3 and L4. However, monosynaptic spiking was rare, and occurred primarily in L4 and L5 non-pyramidal cells. By contrast, during brief, TC-induced UP states, spiking was dense and occurred primarily in pyramidal cells. These network events always involved infragranular layers, whereas involvement of supragranular layers was variable. During UP states, spike latencies were comparable between infragranular and supragranular cells. These data are consistent with a model in which activation of auditory cortex, especially supragranular layers, depends on internally generated network events that represent a non-linear amplification process, are initiated by infragranular cells and tightly regulated by feed-forward inhibitory cells.

## Introduction

For audition, somatosensation and vision, sensory information from the outside world is routed almost entirely through the thalamus, and is projected into neocortex via thalamo-cortical (TC) afferents. Classical experimental approaches to understanding how neocortex uses this information to construct perception relied on single cell recordings under passive (often unconscious) stimulation conditions, emphasizing bottom-up information streams and portraying neocortex as a passive receiver of sensory information (Mountcastle et al., 1957; Hubel and Wiesel, 1963; Gerstein and Kiang, 1964).These approaches provided enormous advances in understanding the hierarchical, columnar and topographic organization of neocortex (Creutzfeldt, 1977; Mountcastle, 1997; Kaas and Collins, 2001). In developing our current understanding of how the cortico-thalamic network constructs awareness, new emphasis is being placed on the pre-eminence of intracortical network activity that is perturbed periodically by volleys of ascending information (Bastos et al., 2012; Singer, 2013).This view is more consistent with the observations that perception depends heavily on internally-generated processes such as expectation, attention and arousal (Warren, 1970; Davis and Johnsrude, 2007; Fritz et al., 2007), that ascending afferents account for a small fraction of synaptic connections in neocortical circuits (Benshalom and White, 1986; Peters and Payne, 1993; Budd, 1998; Schoonover et al., 2014), and that cortical responses to sensory stimuli exhibit a high degree of trial-by-trial variability, due largely to variable cortical activity levels at the time of stimulation (Kisley and Gerstein, 1999; Lakatos et al., 2005; Curto et al., 2009; Pasley et al., 2009; White et al., 2012; Goris et al., 2014).

Nearly ubiquitous in classical descriptions of neocortical function, the “canonical microcircuit model” sought to capture the sequential activation of cells in a neocortical column driven by bottom-up input following a sensory stimulus. As originally conceived, the model consisted of a supragranular network mediating horizontal and ascending cortical-cortical connections, and an infragranular network sending descending cortico-cortical and subcortical projections (Douglas et al., 1989; Douglas and Martin, 1991). In later refinements of the model, based primarily on anatomical data in visual cortex of cat (Gilbert, 1983; Gilbert and Wiesel, 1983; Binzegger et al., 2004) and supported by data from paired recordings in cortical brain slices of rat and cat (Thomson and Bannister, 1998, 2003; Feldmeyer et al., 2002; Thomson et al., 2002; Lübke et al., 2003; Kampa et al., 2006; Thomson and Lamy, 2007), the model predicts a stereotyped pattern of information flow through cortex upon sensory stimulation: spiny stellate cells, excitatory interneurons in layer 4, are activated first by thalamic input, in turn driving ascending projection cells in supragranular layers, which drive descending projection cells in infragranular layers (Douglas and Martin, 2004; Hirsch and Martinez, 2006; Lubke and Feldmeyer, 2007).

In spite of its widespread acceptance, there is evidence that cortical spiking following sensory stimulation is governed by processes in addition to those described in this model. Although electrophysiological mapping studies have shown stronger synaptic strength and higher connection probability from L2/3 to L5 than the reverse (Thomson and Bannister, 1998), such data do not take into account the relative likelihood that cells will spike in response to sensory stimulation, i.e., the observation that L2/3 cells fire far more sparsely than cells in L5 (Sakata and Harris, 2009; Barth and Poulet, 2012), or that infragranular cells often fire with latencies as short or shorter than granular and supragranular cells (Maunsell and Gibson, 1992; Sugimoto et al., 1997; Shen et al., 1999; De Kock et al., 2007; Sakata and Harris, 2009; Christianson et al., 2011; Constantinople and Bruno, 2013; Sun et al., 2013). Indeed, most cortical cells have dendrites that traverse the thalamo-recipient layers, and thus may be activated monosynaptically (Bullier and Henry, 1979; Mitani and Shimokouchi, 1985; Douglas and Martin, 1991; Gil and Amitai, 1996; Verbny et al., 2006; Sun et al., 2013).

Other evidence suggests that features of the model may not apply to auditory cortex in particular. The canonical microcircuit was developed based on studies of visual cortex, where thalamic input contacts spiny stellate cells in layer 4 (Gilbert and Wiesel, 1983). However, spiny stellate cells are rare in auditory cortex, their role likely being occupied by pyramidal cells (Smith and Populin, 2001; Barbour and Callaway, 2008; Sakata and Harris, 2009). Additionally, evidence for receptive field hierarchy from granular to supragranular to infragranular layers is weak compared to visual cortex and is not associated with longer latencies in the infragranular layers (Atencio et al., 2009).

In contrast to the orderly progression of mono-, di- and tri-synaptically-driven spiking activity in layers 4, 2/3, and 5, respectively, predicted by the canonical microcircuit model, spiking in neocortex during sensory stimulation also occurs in the context of synchronous network bursts (Petersen et al., 2003; Deweese and Zador, 2006; Sakata and Harris, 2009). It should be noted that in auditory cortex *in vivo*, there is some debate about these network events, with some authors referring to them as “bumps” (Hromadka et al., 2013) and others as “UP states” (Sakata and Harris, 2009); for simplicity, we will refer to these events as UP states, but we note that there is some controversy regarding this terminology. Although most commonly observed during slow-wave sleep and certain types of anesthesia (Steriade et al., 1993), UP states may share some characteristics with the aroused state, including higher levels of ongoing activity (both excitatory and inhibitory) and depolarized membrane potentials (Steriade et al., 2001; Destexhe et al., 2003, 2007). Indeed, evidence suggests that cortical processing of sensory information occurs during similar, albeit briefer, events in the awake brain (Bathellier et al., 2012; Hromadka et al., 2013; Luczak et al., 2013; Tan et al., 2014). In this model, sensory stimuli trigger these network events with probability that depends on stimulus intensity and identity, in contrast to traditional views of sensory coding in which individual cells' response magnitudes are smoothly varying functions of stimulus properties. Although these events are regulated by subcortical inputs (Metherate et al., 1992; Goard and Dan, 2009; Constantinople and Bruno, 2011), similar activity has also been observed in auditory, visual and somatosensory brain slice preparations (Metherate and Cruikshank, 1999; Sanchez-Vives and McCormick, 2000; Cruikshank et al., 2002; MacLean et al., 2005; Watson et al., 2008; Rigas and Castro-Alamancos, 2009), where it has been shown to be non-epileptiform in nature and represent an *in vitro* correlate of UP states that occur *in vivo* (Sanchez-Vives and McCormick, 2000; Shu et al., 2003; Cunningham et al., 2006; Rigas and Castro-Alamancos, 2007). UP states likely arise in layer 5 before spreading to other laminae (Chauvette et al., 2010; Wester and Contreras, 2012; Beltramo et al., 2013; Stroh et al., 2013), and may represent an intracortical filter that regulates incorporation of sensory signals into the cortical hierarchical processing stream (MacLean et al., 2005). Selective activation of infragranular layers by sensory input (Constantinople and Bruno, 2013) and failure of some UP states to propagate to supragranular layers (Sakata and Harris, 2009) suggests that full engagement of the cortical column may only occur in certain contexts. Here, we present data consistent with a model in which activation of sensory neocortex, especially cells in supragranular layers, depends on internally generated network events initiated by infragranular cells, a process likely tightly regulated by monosynaptic activation of feed-forward inhibitory cells.

## Materials and methods

All experimental protocols conformed to American Physiological Society/National Institutes of Health guidelines and were approved by the University of Wisconsin Animal Care and Use Committee.

### Slice preparation

Male B6CBAF1/J mice (first generation cross of C57BL/6J and CBA/J) were used in these studies, as they represent genetically identical animals that lack recessive mutations known to affect sensory systems (Dräger and Hubel, 1978; Johnson et al., 1997). Mice (3–10 weeks, median 31 days old) were decapitated under isoflurane anesthesia, and their brains were extracted and immersed in cutting artificial CSF [cACSF; composed of (in mM) 111 NaCl, 35 NaHCO_3_, 20 HEPES, 1.8 KCl, 1.05 CaCl_2_, 2.8 MgSO_4_, 1.2 KH_2_PO_4_, and 10 glucose] at 0–4°C. HEPES was included to improve slice health and prevent edema (MacGregor et al., 2001). Auditory TC brain slices (450 μm) were prepared from the right hemisphere as previously described (Cruikshank et al., 2002; Verbny et al., 2006). Slices were maintained in cACSF saturated with 95% O_2_/5% CO_2_ at 24°C for >1 h before transfer to the recording chamber, which was perfused at 3–6 ml/min with ACSF [composed of (in mM) 111 NaCl, 35 NaHCO_3_, 20 HEPES, 1.8 KCl, 2.1 CaCl_2_, 1.4 MgSO_4_, 1.2 KH_2_PO_4_, and 10 glucose] at 30–34°C. Modified ACSF with elevated concentrations of divalent cations used in some calcium imaging experiments as described below was composed of 105 NaCl, 35 NaHCO_3_, 20 HEPES, 3 KCl, 4 CaCl_2_, 4.2 MgCl_2_, and 10 glucose. Auditory cortex was identified based on its position relative to the hippocampus, strong granular layer responses to stimulation of thalamic afferents, and in preliminary experiments by the location of cells retrogradely labeled from the inferior colliculus, as in previous studies (Verbny et al., 2006; Banks et al., 2011). Cortical layers were identified by differences in cell density and based on distance from the pia in conjunction with previous studies (Banks et al., 2011). Afferents were activated using pairs of tungsten electrodes (0.1 MΩ, 75 μm diameter; FHC Inc., Bowdoin, ME). Stimuli (100 μs, 10–150 μA) were applied using constant current stimulus isolation units (A365, WPI Inc., Sarasota, FL; or STG4002, Multichannel Systems, Reutlingen, Germany) and consisted of either single pulses or brief trains (2–4 pulses, 40 Hz). Extracellular recordings in layer 4 taken at 200–300 μm intervals were used to locate the region of auditory cortex best activated by the stimulus and all further extra-/intra-cellular recording and calcium imaging was performed in this region. We followed the well-described procedure for preparing auditory TC slices, and based on the appearance of the slices and the published stereotaxic coordinates of auditory cortex we are confident that all recordings presented here are from auditory cortex. However, the slicing procedure was originally described for juvenile animals (Cruikshank et al., 2002) in which connections between MGv and core auditory cortex could be maintained. Developmental studies have shown that the circuit and membrane properties of neocortex are not fully developed in juvenile animals (Metherate and Aramakis, 1999; Frick et al., 2007; Oswald and Reyes, 2008, 2011; Romand et al., 2011), consistent with observations *in vivo* showing developmental changes in tuning properties of auditory cortical neurons between 3 and 5 weeks of age (Chang and Merzenich, 2003; Chang et al., 2005). Thus, we confined our study to animals >3 weeks old, and in most cases >4 weeks old, in which the connections from MGv to core auditory cortex were not always intact. To activate auditory TC fibers in slices from older animals, we stimulated outside of MGv, just rostral to the nucleus or in the fiber bundle (the superior thalamic radiation) that runs from auditory thalamus to auditory cortex. We verified that stimulation of these fibers elicited current sinks in layer 4 in all experiments. However, we cannot exclude the possibility that some of our recordings were from areas outside of A1 that receive driving core auditory thalamic inputs, e.g., AuV or AuD in the Paxinos (Paxinos and Franklin, 2003) terminology. Indeed, given that the slices are 450 μm thick, even if we were to place stimulating electrodes in MGv and record responses in auditory cortex, it cannot be guaranteed that all the cells recorded are located in core auditory cortex and that we are only stimulating fibers arising in MGv.

### Electrophysiological recordings

Electrophysiological recordings were obtained using patch pipettes fabricated from borosilicate glass (KG-33; 1.7 mm outer diameter; 1.1 mm inner diameter; Garner Glass, Claremont, CA) with a Flaming-Brown two-stage puller (P-87; Sutter Instruments, Novato, CA). For whole-cell current clamp recordings, pipettes had open tip resistances of 3–5 MΩ when filled with (in mM): 140 K-gluconate, 10 NaCl, 10 HEPES, 0.1 EGTA and 2 MgATP, 0.3% biocytin, pH 7.2. Cells were visualized using a video camera (Dage MTI VE-1000) connected to an upright microscope (Olympus BX51-WI) with a long working-distance water-immersion objective (Olympus 40X, 0.9 N.A.) and differential interference contrast optics. For extracellular field potentials, the tips of the pipettes were broken under visual control to an outer tip diameter of 10–15 μm and had open-tip resistances of about 0.5 MΩ when filled with ACSF. Data were amplified (MultiClamp-700A; Molecular Devices, Union City, CA), low-pass filtered (4 kHz), digitized (40 kHz; DigiData 1322A; Molecular Devices), and recorded using pClamp version 9.2 (Molecular Devices). Current source density (CSD) and multiunit activity (MUA) was derived from extracellular recordings in which we recorded local field potentials simultaneously in all cortical layers using 16-channel silicon electrode arrays (A-series probes; 16 shanks spaced by 100 μm, one recording site per shank; 1 MΩ impedance; NeuroNexus, Ann Arbor, MI) connected to a unity-gain headstage (HS-16; Neuralynx, Bozeman, MT), amplified 2000× (Lynx-8, Neuralynx), digitized at 20 kHz (Digidata 1440A; Molecular Devices, Sunnyvale, CA) and recorded using pClamp (Molecular Devices). The electrode array was inserted into the slice at an acute angle with the shanks oriented in parallel to the cortical laminae. Depending on the cortical thickness at the recording site, 11–13 of the electrode shanks were in cortex and used in calculating the CSD and MUA.

### Calcium imaging

Calcium imaging using the acetoxymethyl ester form of Oregon Green BAPTA-1 (OGB-1 AM; Life Technologies, Grand Island, NY) was performed as described previously (Banks et al., 2011). Briefly, patch pipettes of ~2 μm outer tip diameter were filled with OGB-1 AM dissolved in DMSO containing 20% pluronic acid to a dye concentration of 5 mM and then gradually diluted using extracellular solution containing (in mM) 150 NaCl, 2.5 KCl, 10 HEPES, pH 7.4 to a final dye concentration of 0.165 mM. The tissue was loaded using pressure ejection (Picospitzer II, General Valve, Fairfield, NJ; 5–10 PSI, 1–10 s pulses over 1–2 min) at 5–7 locations over a period of ~45 min. The total area labeled in this way measured ~200 × 800 μm. Data collection commenced ~60 min following the last injection. Data were collected using an upright microscope (BX51-WI, Olympus, Center Valley, PA), mercury arc lamp light source (X-Cite exacte; Lumen Dynamics, Mississauga, Ontario, Canada) with an excitation filter 475–505 nm and emission filter 520–570 nm (U-N41026, Chroma, Bellows Falls, VT), and a 10× water immersion objective (UMPlanFL N, N.A. = 0.3, Olympus). Images were captured at 30 frames/sec with a cooled CCD camera (500 × 500 pixels, corresponding to 800 × 800 μm, 16-bit; C9100-02, Hamamatsu Corp., Sewickley, PA) using SimplePCI software (v6.1, Hamamatsu). For calcium imaging experiments (except when multiple stimulus intensities are shown), the stimulus intensity was set high enough to trigger UP states under normal ACSF on consecutive trials using four pulses at 40 Hz and low enough that UP states were blocked using high divalent ACSF, typically 50–100 μA.

### Data analysis

#### Electrophysiological recordings

EPSP latencies and amplitudes were obtained from averages of ten responses to single 100 μA pulses. EPSP latencies were measured as the time of rise to 10% of the peak. EPSP amplitude was measured as rest-to-peak and cells were omitted from amplitude measurement if they had a coincident IPSP revealed by depolarizing the cell. IPSPs were identified by stimulating while holding the cell at depolarized potentials using small current steps up to spike threshold. Step size varied with the input resistance, resting potential, and spike threshold of the cells as necessary to result in 5–10 steps between rest and threshold. Spike thresholds were measured as the membrane potential at the peak of the 2nd derivative of the voltage trace. Spike latencies were measured as time to peak. Monosynaptic spikes were those that occurred in response to a smooth, putative monosynaptic EPSP with short latency and low latency jitter (EPSP latency <5 ms, *SD* < 1 ms; spike latency <10 ms, *SD* < 1 ms) (Berry and Pentreath, 1976; Rose and Metherate, 2005). We note that the actual EPSP latency jitter preceding monosynaptic spikes was much more precise than this criterion (jitter mean ± *SD*: 0.23 ± 0.21 ms).

UP states were detected using the MUA signal obtained in extracellular recordings (using multichannel electrodes or glass field potential electrodes) from layer 5 using a method similar to that of Sakata and Harris (2009). We extracted the MUA signal by bandpass filtering the voltage signal between 0.5 and 3 kHz, taking the absolute value, and then smoothing with a lowpass filter (0.2 kHz cutoff) to yield the smoothed MUA signal, smMUA (t). A threshold was determined by computing the geometric mean *x* of smMUA (t) on data points greater than the mean of smMUA (t) during the pre-stimulus period. UP state onset was defined as the time at which smMUA (t) >*x* for 80% of the points in a 20 ms window, and offset was defined as the subsequent time when smMUA (t) <*x* for 80% of the points in a following 20 ms window. For multichannel recordings, CSD (Freeman and Nicholson, 1975; Mitzdorf, 1985) was estimated using the spline CSD method (Pettersen et al., 2006). MUA in the form of single action potentials was extracted from these same multichannel recordings by bandpass filtering the extracellular signal between 0.5 and 3 kHz and detecting negative-going level crossings using a threshold of 5 times the standard deviation of the baseline period.

We sought to compare directly the observed pattern of spiking responses observed via calcium imaging to the pattern we observed in on-cell recordings, in which we measured the number of spikes fired for each cell over a series of 10 trials. To compare these spiking probabilities to the calcium imaging data, we converted the spiking probability to the likelihood to detect a cell according to our calcium imaging calibration data illustrated in **Figure 8F** (using linear interpolation when necessary) and averaged across cells in each layer.

All statistical analyses of electrophysiological data used native MATLAB (Mathworks, Natick, MA) functions. Non-parametric tests were used when data were distributed significantly different from the normal distribution (one-sample Kolmogorov–Smirnov test, *p* < 0.05). When multiple pairwise comparisons were performed, significance thresholds were adjusted using the Holm–Bonferroni method (nominal α = 0.05); *p*-values were not adjusted.

#### Calcium imaging

Fluorescence traces (reported as ΔF/F, *not %Δ*F*/*F**) were analyzed as described (Banks et al., 2011). Briefly, ΔF/F as a function of time was measured in single cells identified in averaged still images using custom software written in MATLAB. Traces were background-subtracted and peaks corresponding to action potential activity were detected. Cells were identified as “responsive” when the sustained peak increase in fluorescence (over a window of 4 data points or 133 ms) was greater than 3 times the standard deviation of a 1-s baseline before stimulation. In some experiments, we targeted responsive cells identified via calcium imaging for subsequent whole-cell patch clamp (see Results). Spikes were detected in all recordings of these cells, confirming that observed calcium transients were associated with action potentials. We computed the laminar spiking profile within a single experiment (e.g., **Figure 7D**) by dividing the cortical depth into 50 μm bins and counting the number of cells in each bin. When data were pooled across experiments (e.g., **Figure 10**), bin size was expanded to 100 μm to make plots easier to read. All analyses presented here were insensitive to this change in bin size. To compare relative dye (OGB-1) levels across analysis windows, the average baseline fluorescence was integrated across space and normalized to the bin with the largest integrated baseline fluorescence from that experiment. By using an integral rather than a spatial average, this measure incorporates variability in background (labeling intensity) due to heterogeneous loading as well as depth bins that fell outside the square imaging window (particularly layers 1 and deep layer 6).

Statistical comparisons between layers and between bathing media (normal ACSF or ACSF modified with high divalent cations or APV) were made by fitting a Poisson, log-linked Generalized Estimating Equations (GEE) model to cell count measures normalized by layer thickness (Reed and Kaas, 2010). A standard ANOVA or general linear model (GLM) approach is less appropriate because the cell count data are not normally distributed, resulting in poor error estimates, especially when some counts are very low or zero. GEE models are suitable for repeated measures data (here, repeated across layers in each experiment and within layers for each ACSF condition) in which population effects are of interest but within-subject prediction is not necessary. Because of the logarithmic link function, a linear combination of parameters results in a multiplicative effect on the outcome data, in this case cell count. When comparing level parameters, we computed the Wald test on the parameter estimates themselves, but then present the exponent of the parameter, e.g., as “exp(β_Condition_),” and its confidence interval because this exponent is an easily understood multiplicative factor to the cell counts. Significance thresholds were adjusted using the Holm–Bonferroni method as above.

The GEE model was fit using IBM SPSS Statistics 22. *Post-hoc* tests and all other analyses used native and custom MATLAB functions.

### Histology

Slices containing biocytin-filled cells were carefully removed from the recording chamber and immediately fixed in freshly prepared 4% paraformaldehyde solution. After at least 24 h in fixative, slices were sectioned and processed for biocytin staining using the cobalt/nickel intensification method (Adams, 1981). First, the slice was cryoprotected by passage through a series of glycerol–sucrose solutions. Labeled cells were often close to the slice surface so to assure that this tissue was not lost in the sectioning process the following method was used. The slice was placed on a glass slide and covered with a drop (s) of a 4% paraformaldehyde (1 ml)/egg albumin (10 ml) solution. A second slide was gently laid over the slice to flatten it. After the albumin had solidified the slides were removed and the flattened albumin-embedded slice was mounted on a freezing microtome and 60 or 90 μm sections were cut and collected in 0.1 M phosphate buffer (pH 7.4). Sections were then washed in 0.01 M phosphate buffered saline (PBS, pH 7.4), incubated in 0.5% H_2_O_2_ in PBS, and rinsed again in 0.01 M PBS. Sections were then incubated overnight in the secondary antibody to the avidin-biotin-HRP complex (ABC Kit, Vector Labs) in PBS with 0.3% Triton-X-100 and 2% BSA. On the second day, the sections were rinsed in 0.1 M PBS and the HRP was reacted using 0.04% diaminobenzidine with DMSO and nickel/cobalt intensification. We then added 0.01% H_2_O_2_ in 0.1 M PBS for 15 min followed by rinsing for 40 min in PBS. The sections were then mounted on slides, allowed to air dry and the mounted sections were then stained with cresyl violet and coverslipped. Photomicrographs of labeled cells were taken at 5, 25, and 100×. For the image in **Figure 4E**, the major processes of the labeled cell were found in two tissue sections. To overlay the images and produce a unified picture, the position of the superficial section relative to the deeper section was determined by aligning the 5X images with common structures. The Eyedropper tool in Adobe Photoshop was used to create a color mask to remove the background and Nissl-stained cells from the superficial section, leaving the darkly labeled processes, and this image was merged with the unaltered deep image section. The “auto-contrast” function in Photoshop was used on both photomicrographs in **Figure 4** to improve the differentiation between the Nissl-stained cell bodies in the background and the biocytin-labeled somata and processes of interest. All image processing steps were performed across the entire image and did not alter the information contained.

## Results

### Overview

The data presented here were obtained to answer a simple question: what is the pattern of spiking elicited in auditory cortex by stimulation of core thalamic afferents? In classical models of the cortical microcircuit, TC afferents terminating in layer 4 activate excitatory interneurons (spiny stellate cells) resident in that layer, and spiking activity in these cells triggers sequential activation of pyramidal cells and interneurons in supragranular and then in infragranular layers. In primary auditory cortex, where spiny stellate cells are rare or absent (Smith and Populin, 2001; Barbour and Callaway, 2008), it is unclear whether this activation sequence is preserved. To address this issue, we recorded from 103 cells selected at random throughout layers 2–6 (L2/3 = 21, L4 = 19, L5 = 51, L6 = 12) using whole-cell patch clamp, and 104 cells selected at random throughout layers 2–6 (L2/3 = 27, L4 = 22, L5 = 47, L6 = 8) using on-cell recordings. We focus first on short latency, putatively monosynaptic responses, showing that spiking directly in response to these inputs is rare. We then show that spiking is common in the context of induced UP states and examine the laminar profile of this activity. We corroborate these findings from single cell recordings using two techniques that allow us to monitor spiking activity simultaneously across the cortical laminae: multichannel recordings of MUA (*n* = 16 slices) and calcium imaging using bulk loading of neurons with OGB-1AM (*n* = 1588 cells in 17 slices).

### Laminar profile of current sinks activated by TC stimulation

**Figure 1:**
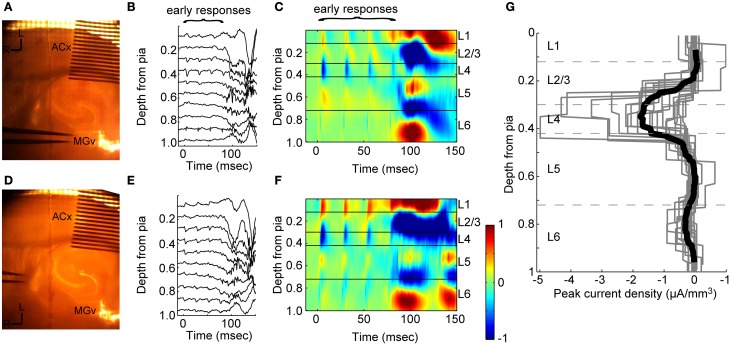
**Extracellular responses to stimulation of thalamic afferents. (A)** Experiment setup for CSD recordings. A 16-shank electrode is inserted into auditory cortex (100 μm between shanks) and a bipolar stimulating electrode is located in MGv. **(B)** Example filtered (1–300 Hz) LFP from one trial in response to four stimulus pulses at 40 Hz. Stimulus onset at *t* = 0. **(C)** CSD for this experiment calculated from an average of 20 trials as in **(B)** shows the laminar pattern of current sinks (*blue and cold colors*) and sources (*red and hot colors)* elicited by stimulation in MGv. Early current sinks are located in layer 4 and deep layer 3 (“early responses”). These early sinks are followed by large current sinks in both infragranular and granular layers which we refer to as UP states (see Figure 2). The vertical depth axis is normalized to the distance from the pia to white matter, typically around 1 mm for mouse auditory cortex. Color scale is ±1 μA/mm^3^. **(D–F)** Same experiment as in **(A–C)**, but with stimulating electrodes placed in the superior thalamic radiation containing thalamo-cortical fibers instead of directly in MGv, as was common in experiments on older animals. Early and late responses were nearly identical between TC fiber stimulation and stimulation in MGv. **(G)** Peak negative current density in the window 3 < *t* < 20 ms after the first thalamic stimulus pulse for individual experiments (gray lines) shows the initial monosynaptic component is located in layer 4 and deep in layer 3 (dark black line: mean across experiments). Dashed lines indicate layer boundaries.

We first verified that stimulation of TC afferents resulted in the expected laminar profile of synaptic current flow using multichannel recordings (Figure 1A). TC stimulation resulted in an early, large, fast sink (“early responses,” LFP Figure 1B; CSD Figure 1C; time to 10% of peak = 2.0 ± 1.1 ms, *n* = 16 slices) located in layer 4 (mean depth = 36 ± 5% of cortical depth) that sometimes extended into layer 3, as expected based on the laminar distribution of terminals in primary auditory cortex arising from MGv (Huang and Winer, 2000; Smith and Populin, 2001; Smith et al., 2012). There was usually an additional, weaker sink observed simultaneously in layer 6, which also receives direct MGv input. We typically observed modest depression of current sinks evoked by TC stimulation trains as seen previously with TC EPSPs (Rose and Metherate, 2005; Lee and Sherman, 2008).

Most of the data presented here were obtained in animals >4 weeks old, in which it is difficult or impossible to obtain slices with intact fiber bundles between MGv and auditory cortex. Thus, in most experiments stimulating electrodes were placed just rostral to the MGv, where the TC fibers coalesce, or in the superior thalamic radiation, just rostral and medial to hippocampus. In four experiments from 3 to 4 week old animals, we compared directly the current sinks obtained by stimulating in these locations with those obtained by stimulating in MGv. The results of one of these experiments are illustrated in Figure 1. In the same slice illustrated in Figures 1A–C, we moved the stimulating electrode to the fiber bundle just rostral to the hippocampus (Figure 1D) and obtained similar LFP/CSD patterns (Figures 1E,F) as those elicited by direct MGv stimulation (Figures 1B,C). Similar results were obtained in all four experiments in which we compared directly stimulation in MGv, just rostral to MGv, and in the fiber bundle rostral to hippocampus. Note that larger current sinks could be obtained by stimulating in the superior thalamic radiation compared to MGv itself. These results suggest that stimulation in the fiber bundle in older animals elicited responses that are equivalent to stimulating in MGv itself.

We summarize the results of 16 such recordings in older animals in Figure 1G, which shows the laminar profiles of current sinks evoked by TC stimulation. To compare across slices with slightly varying cortical depths, we normalized the single experiment profiles by dividing by the distance from the pia to white matter. These recordings clearly show that the vast majority of synaptic current flow in response to TC stimulation occurs in layers 3 and 4, with a modest response component in layer 6. In none of these experiments were early current sinks observed in layers 1, 2, or 5, indicating that activation of “matrix” (i.e., non-specific or extralemniscal) thalamic afferents in our stimulation protocol was minimal.

### Brief UP states triggered by TC stimulation

**Figure 2:**
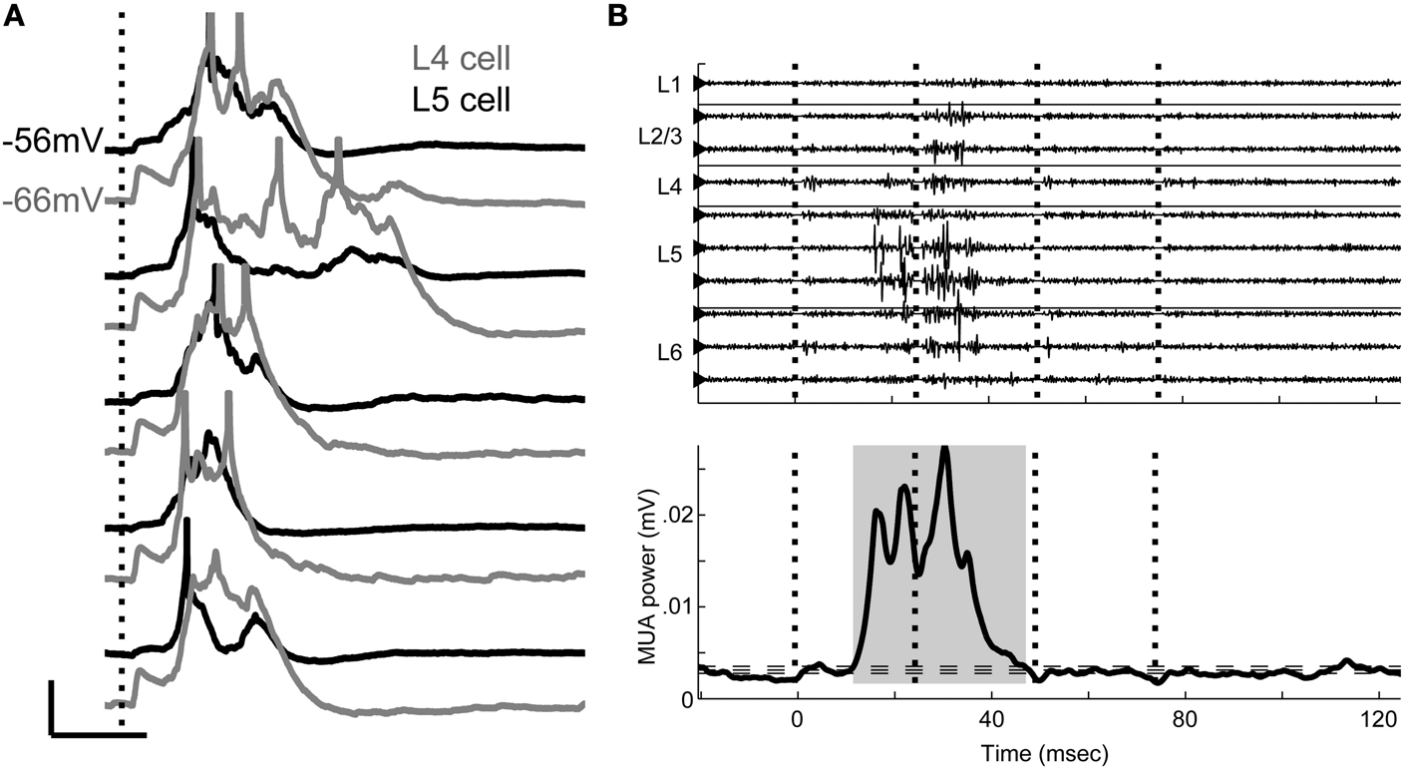
**Evoked UP states. (A)** Simultaneous whole-cell recordings from a layer 4 cell (gray) and a layer 5 cell (black) on five consecutive trials in response to single stimulation pulse applied to TC afferents (vertical dotted line) show that UP states are network events with variable duration between trials but consistent time course between cells on a given trial. Scale bar: 50 ms, 10 mV. Action potentials are truncated. **(B)** Multiunit activity (filtered 0.3–3 kHz) from multichannel recordings similar to Figure 1 shows the UP state as a burst of high frequency activity occurring on multiple channels, but beginning in layer 5 (*top*). Vertical dotted lines mark stimulus times. The smoothed MUA power in layer 5 is used to detect UP states (shaded gray area, *bottom*) on single trials. Horizontal dashed lines represent the boundaries used to detect UP state onset and offset (see Materials and Methods).

In addition to early response components, TC stimulation typically triggered network events, seen in CSD profiles (Figures 1C,F) as intense patterns of sources and sinks subsequent to the initial TC response. During these events, cells throughout the cortical region depolarized nearly simultaneously under a barrage of synaptic inputs (Figure 2A), and occasionally fired action potentials. We also observed spontaneous UP states in 5/10 slices examined, but at low rates (0.2–2 per minute). Between these occasional events, the slice was in a persistent DOWN state, as is typical of cortical brain slices (Sanchez-Vives and McCormick, 2000).

The UP state activity appeared in extracellular recordings in layer 5 as a low frequency negative or sometimes biphasic wave with associated high frequency (spiking) activity (Figure 2B, *top*) that often spread to involve all cortical layers, as previously described *in vitro* and *in vivo* (Sanchez-Vives and McCormick, 2000; Luczak et al., 2007; Sakata and Harris, 2009; Chauvette et al., 2010; Wester and Contreras, 2012; Beltramo et al., 2013). Following the method of Sakata and Harris (Sakata and Harris, 2009) for data recorded *in vivo*, we detected these events using the MUA signal derived from extracellular recordings in layer 5 (Figure 2B, *bottom*; see Materials and Methods). Below, we show that although synaptic responses to TC afferents are widespread in auditory cortex, it is rare for cells to spike in direct response to these EPSPs. Rather, the vast majority of spiking in response to TC stimulation occurs instead in the context of these UP states.

### Short latency responses to TC stimulation

**Figure 3:**
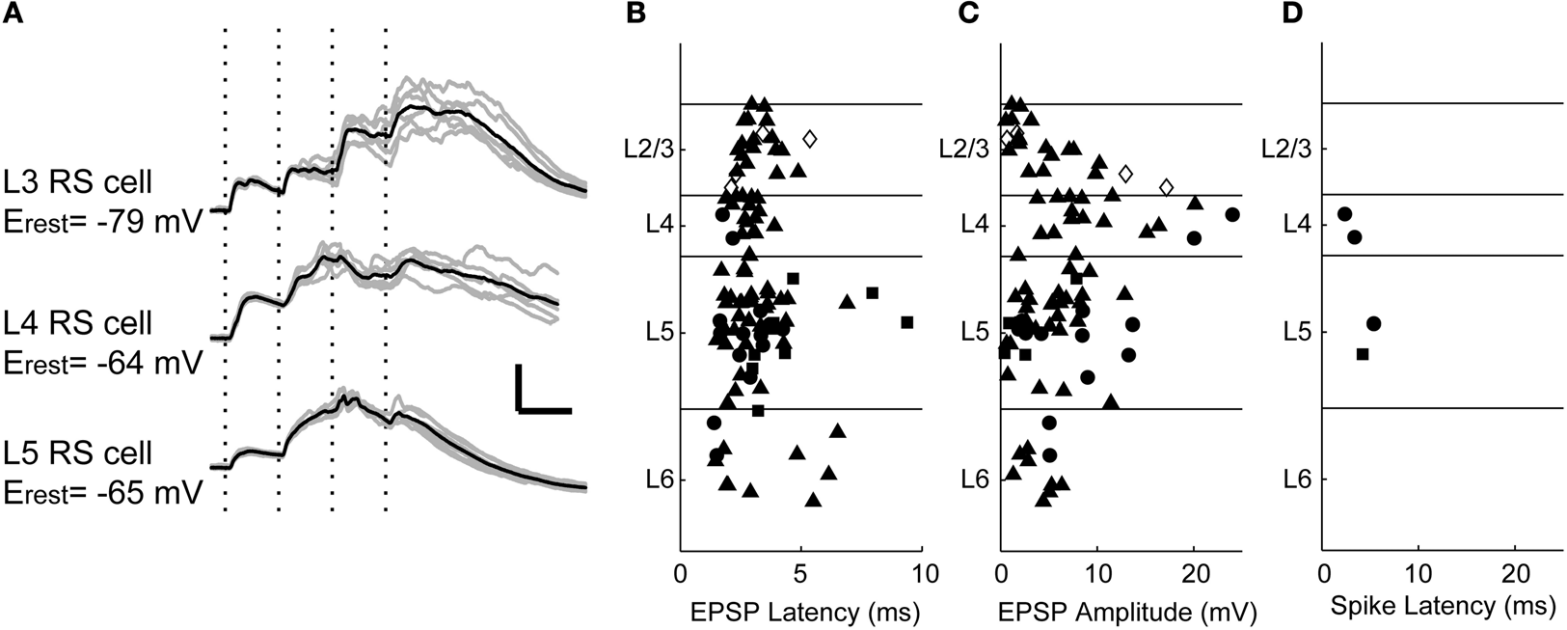
**Intracellular responses to TC stimulation. (A)** EPSPs evoked by TC stimulation (four 100 μA pulses at 40 Hz, marked with vertical dotted lines) in regular spiking cells of layers 3–5. Polysynaptic activity associated with UP states is observed in all three cells after the second stimulus. Gray traces are five consecutive single trials for each cell, black traces are averages. Scale bar: 25 ms, 10 mV. **(B)** Shortest EPSP latency to TC stimulation (100 μ A) was consistent across layers. Symbols represent cell types identified based on spiking patterns to depolarizing current (*filled triangle* = regular spiking, *filled circle* = fast-spike or burst-firing interneurons, *filled square* = intrinsically bursting, *open diamond* = unclassified). **(C)** EPSP amplitudes plotted with the same conventions and stimulus as in **(B)**. **(D)** Short latency spikes after a single stimulus pulse were rare. In only four randomly targeted cells were monosynaptic thalamo-cortical inputs superthreshold. Three of these cells were fast-spiking non-pyramidal cells, and one was an intrinsically bursting pyramidal cell (see Figure 4).

Using whole-cell patch clamp recordings, we examined the intracellular responses to stimulation of TC afferents in 103 cells (*n* = 21, 19, 51, and 12 cells randomly selected from layers 2/3, 4, 5, and 6, respectively; note that because layer 5 is roughly twice as thick as layers 2/3 and 4, this represents a roughly uniform sampling density for layers 2/3 through 5). Although the synaptic terminals of stimulated afferents target specific layers of cortex, we found that TC input triggered short latency EPSPs in cells of all layers (Figure 3A), most likely by synapsing on these cells' dendritic processes that span thalamo-recipient layers. Latencies were similar across the cortical laminae (Figure 3B; median latencies: L2/3, 3.0 ms; L4, 2.9 ms; L5, 3.0 ms; L6, 2.4 ms; no significant difference, Kruskal–Wallis *H* = 2.79, 3 d.f., *p* = 0.43). These short latencies are consistent with nearly all these responses being monosynaptic (Rose and Metherate, 2005; Verbny et al., 2006), except perhaps a few >5 ms in layers 5 and 6; likewise, the similarity of latencies across laminae is inconsistent with EPSPs in layers 2/3 and 5 being di- and tri-synaptic responses driven by intracortical spiking activity. The amplitude distribution of the initial EPSPs was more heterogeneous (Figure 3C; Kruskal–Wallis *H* = 14.79, 3 d.f., *p* = 0.002): the largest EPSPs were observed in layer 4, but we observed large EPSPs (>5 mV) in layers 3–6 (median EPSP amplitudes: L2/3:2.9 mV; L4:8.1 mV; L5: 5.2 mV; L6:4.7 mV; L4 significantly greater than each of the other layers by Tukey HSD).

**Figure 4:**
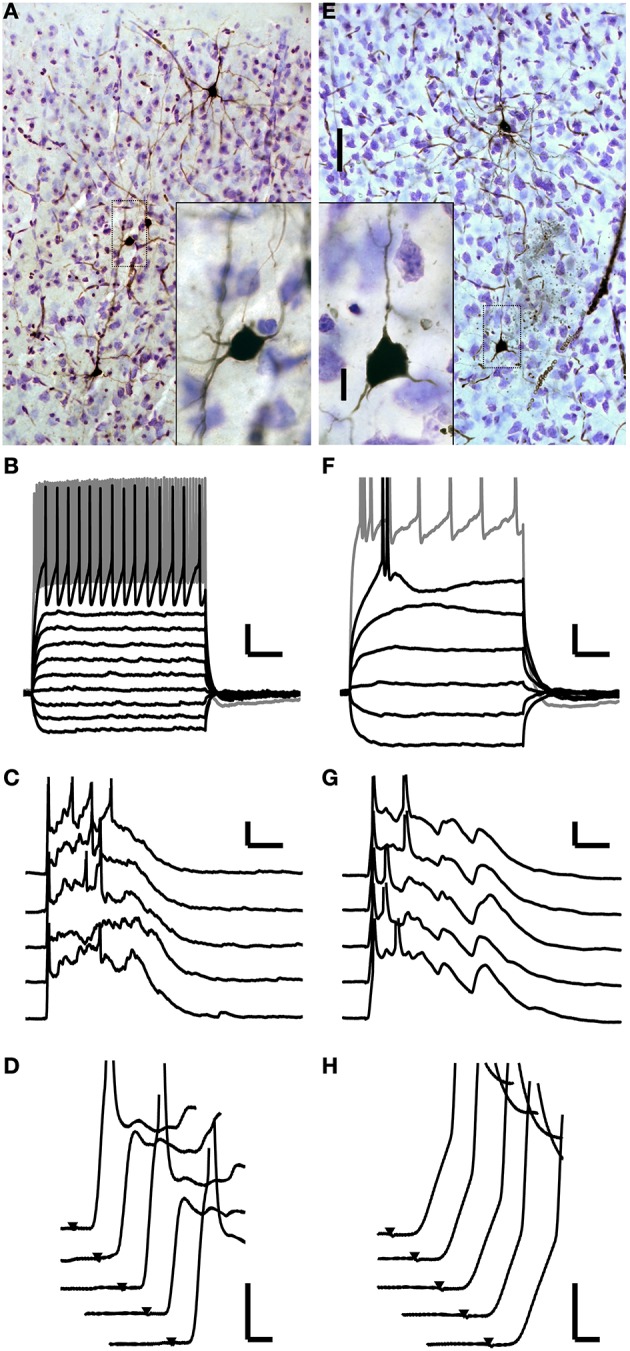
**Monosynaptically activated cells. (A–D)** One of two monosynaptic spiking interneurons in layer 4. **(A)** Photomicrograph of this cell along with others recorded in the same slice. The cell is non-pyramidal and aspiny. The cell of interest is surrounded by a gray box, which demarcates the boundaries of the inset. **(B)** Polarizing current pulses identify this cell as a fast-spiking cell. **(C)** This cell fires early, monosynaptically driven spikes as well as later spikes during evoked UP states. Action potentials are truncated. **(D)** Detailed inspection of the early component of the responses from **(C)** shows that the spikes occur after a smooth initial depolarization with no evidence of di- or poly-synaptic potentials contributing to the early, monosynaptic spikes. This is also clear on trials two and four where the cell did not fire a spike. **(E–H)** A monosynaptic spiking pyramidal cell in layer 5. **(E)** Photomicrograph as in **(A)**. **(F)** This cell has an intrinsically bursting firing pattern. **(G,H)** Similar to the cell shown in **(A–D)** this cell fires early, monosynaptic spikes as well as later spikes during evoked UP states. Scale bars: **A,E** 50 μm (25X), 10 μm (100X); **B,F**: 50 ms, 10 mV; **C,G**: 25 ms, 10 mV; **D,H**: 2 ms, 10 mV.

Although many cells throughout the cortical laminae received monosynaptic TC inputs, spiking in response to these short latency EPSPs was rare. In these 103 cells, spikes in response to putatively monosynaptic thalamic EPSPs triggered by the first TC stimulus were observed in only four cells, two in layer 4 and two in layer 5 (Figure 3D). Both layer 4 cells (one example Figures 4A–D) and one of the layer 5 cells were fast-spiking interneurons. The fast-spiking cells were smooth, non-pyramidal cells (Figure 4A) and fit the classical characteristics of parvalbumin-positive interneurons (Kawaguchi and Kubota, 1997). The fourth monosynaptic-spiking cell was an intrinsically bursting pyramidal cell in layer 5 (Figures 4E–H). We defined these four cells as “monosynaptic spiking” cells based on the EPSPs leading to spikes being short latency (like the EPSPs of Figure 3A) and smooth, without evidence of additional di- or poly-synaptic activity prior to spike onset. We note that we are unable to ascertain without a doubt that these spikes were driven only by monosynaptic inputs, but given their latency and their occurrence prior to induced UP states, these are the only cells we observed that could potentially be driven monosynaptically. In both of the cells pictured, these monosynaptic spikes were followed by UP state activity such as in Figure 2 which was associated with additional spikes driven by polysynaptic activity. In the remaining 99 whole-cell recordings, all cells that spiked following TC stimulation fired late, UP state-associated spikes.

### Spiking responses are dominated by activity during UP states

The observed sparseness of monosynaptic spiking responses to thalamic stimulation is surprising, given the pre-eminence of TC pathways for carrying sensory information to cortex. Because the intracellular milieu is disturbed during whole-cell recordings, it is possible that spike thresholds and spiking probability were altered in the recordings presented above. In 33 cells, we obtained on-cell recordings prior to the whole-cell recordings from Figures 3, 4, including two of the previously described cells that fired monosynaptic spikes according to our definition. In on-cell recordings, both of these cells also fired short-latency (<10 ms) spikes to TC stimulation with high temporal precision (standard deviation of spike times <1 ms) in the on-cell recordings. None of the other 31 cells fired spikes that met these criteria. We also recorded on-cell spiking responses to TC stimulation in 71 additional cells (for a total of 104 on-cell recordings). None of these cells fired spikes that met that <10 ms latency and <1 ms standard deviation criterion. We acknowledge that we would be unable to confidently identify monosynaptic-spiking cells using the on-cell data alone, but emphasize that no cells in the “on-cell only” dataset met even these less stringent criteria.

**Figure 5:**
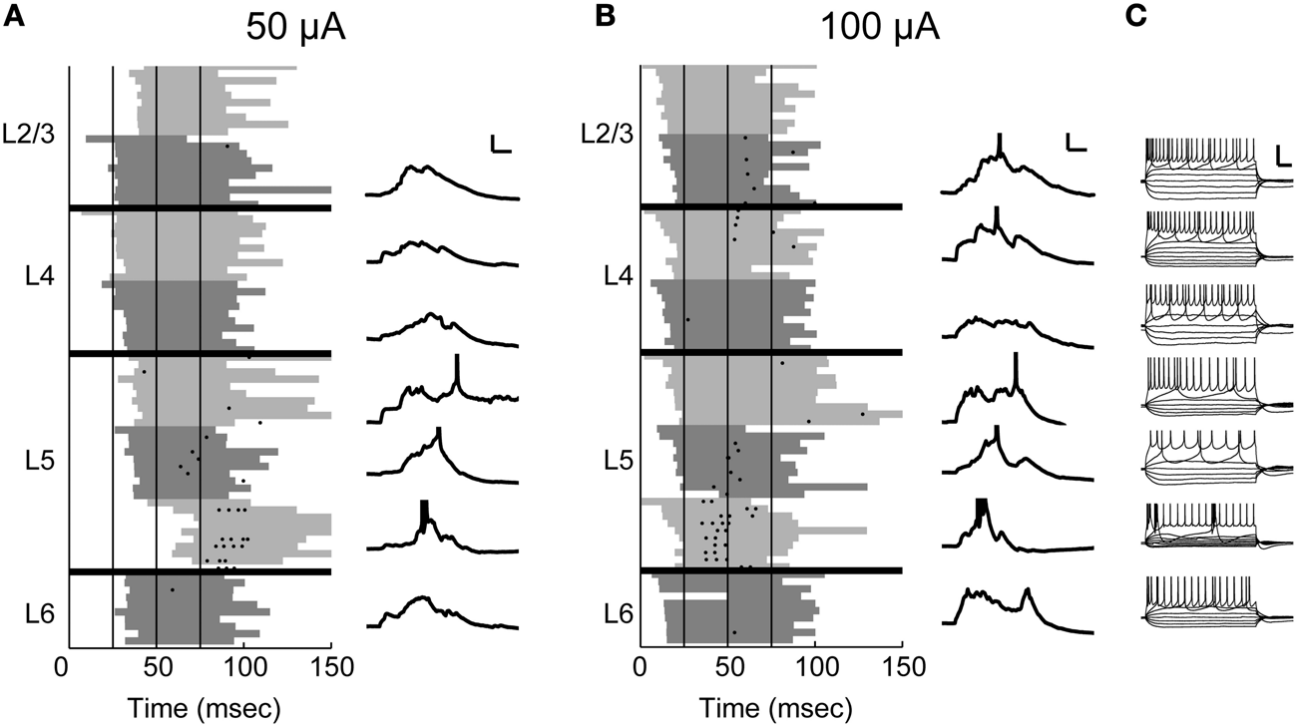
**On-cell spiking at moderate and high stimulus intensities**. In this example experiment, 8 sequential on-cell recordings were obtained from cells across the cortical laminae. **(A)** The recorded cells are plotted in order according to their laminar position. A spike raster of 10 trials is plotted for each cell (*left*). The shaded gray area delineates UP states detected on the multiunit activity recorded on a low-impedance glass electrode placed in layer 5 for the duration of the experiment. The shade of gray used alternates between light and dark for each cell to better distinguish between cells. Vertical black lines indicate stimulus pulses (4 × 40 Hz). At 50 μ A stimulus intensity, UP states are typically evoked after the second stimulus pulse, and on-cell spikes were only detected in the infragranular layers. Whole-cell recordings obtained after the on-cell experiment were available in 7/8 cells (*right*) and confirm that the observed spikes occurred during evoked UP states. Scale bar 25 ms, 10 mV. **(B)** Conventions as in **(A)**, but at a higher stimulus intensity. At this intensity, UP states are evoked after the first stimulus pulse, and there are some spikes in layers 2/3 and 4 as well as more spikes in cells in layer 5. **(C)** Polarizing pulses in whole-cell recordings show that 6/7 cells were regular spiking and the deepest layer 5 cell was intrinsically bursting. Scale bar 50 ms, 30 mV.

Despite the apparent paucity of monosynaptic spiking, TC stimulation consistently elicited robust spiking activity in the context of evoked UP states (Figure 2). To investigate the laminar profile of this spiking activity and its latency distribution, we sequentially and systematically measured spike times during on-cell recordings from randomly targeted cells at different cortical depths in the same slice (*n* = 4–9 cells in each slice) while simultaneously recording extracellularly from layer 5 to monitor the occurrence of evoked UP states. The data from eight cells recorded in one such experiment are illustrated in Figure 5. As was typical for these experiments, all spikes occurred during UP states (gray background) and at substantial delays relative to the first stimulus in the train. At low stimulus intensities (Figure 5A), spikes were observed only in layer 5 cells. Increasing the stimulus intensity (Figure 5B) decreased the latency of UP states and triggered spiking activity additionally in granular and supragranular cells, but the density of spiking was greater for cells in layer 5, and the spike latency in these cells was often shorter than that of cells in layers 2–4. Electrophysiological responses to polarizing current pulses are shown in Figure 5C; most of the cells in this experiment were regular spiking and likely pyramidal; the deepest layer 5 cell was intrinsically bursting.

**Figure 6:**
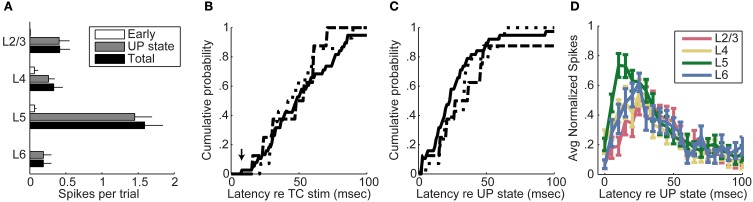
**Timing of spiking relative to UP states. (A)** Average spikes per trial per cell across all on-cell recordings. Across all on-cell experiments, almost all spikes in all layers were fired during UP states. Error bars represent standard error of the mean. Total spike count includes early and UP state spikes as well as additional spikes that did not fit either category (for example, spikes occurring after an UP state, or spikes occurring at long latency in the absence of a detected UP state). **(B)** Cumulative mean first-spike latency distributions relative to stimulus onset show no significant differences between layers 2/3, 4, and 5. The mean first spike latency for each cell was determined by averaging the latency to the earliest spike across trials. Layer 6 is omitted from this figure due to the relatively low number of cells recorded in layer 6. *Dotted line*, Layer 2/3; *Dashed line*, Layer 4; *Solid line*, Layer 5. **(C)** Cumulative mean first-spike latency distributions relative to UP state onset similarly show no significant differences between layers. **(D)** Spike latencies relative to UP states in MUA from multichannel recordings show that layer 5 is active before the other layers during UP states.

We summarize data from all 104 cells in Figure 6A by showing the laminar profile of the spiking activity averaged across cells in each layer. Here we use a more inclusive definition of “early” spikes that included all spikes that came before the onset of UP states and no later than 20 ms after the final stimulus pulse. It is clear from these data that thalamic stimuli are effective at triggering spiking in auditory cortex, but almost all of these spikes occurred during UP states. Almost all early spikes occurred in layers 4 and 5. Spike rates were highest in layer 5, but spikes were detected in all laminae examined. In Figures 6B,C, we have plotted the cumulative distributions of latency relative to stimulus onset (Figure 6B) or relative to UP state onset (Figure 6C) for layers 2/3, 4, and 5. It is apparent that the earliest cells to spike are in layer 5 (Figure 6B, *arrow*), and that majority of cells in layers 2/3 and layer 5 are activated at about the same latency (no significant difference between distributions, Kolmogorov–Smirnov test), both relative to stimulus onset and UP state onset. The high firing rate of layer 5 cells and the fact that early spikes were nearly non-existent in layers 2/3 are more consistent with a model in which layer 5 is directly activated by TC input and subsequently by intralaminar activity, as opposed to being driven by layer 2/3 cells in a sequential layer 4 to layer 2/3 to layer 5 pattern as predicted by the canonical microcircuit.

The advantage of data obtained from patch clamp recordings such as those shown in Figures 5, 6 is that we can identify spikes associated with individual cells, and we can identify the laminar position of the cell body and thus the position of the cell in the cortical microcircuit. However, it was necessary to obtain these recordings sequentially, which could potentially distort the laminar spiking profile by adding variability in latencies that could obscure the progression of latencies in different cell types. To complement these single cell recordings, we also used multichannel MUA recordings to record from large numbers of cells simultaneously across the cortical laminae. To look at relative spiking latency between layers during UP states, we counted spikes in each layer in 5 ms bins after UP state onset, divided by the maximum spikes per bin for that layer for that experiment, and then averaged these normalized spike rates across experiments (Figure 6D). The time of spiking onset, defined as the earliest bin with at least 10% as many spikes as the peak bin, was significantly different between layers [Friedman test, χ^2^_(3)_ = 28.84, *p* = 0.000002]; layer 5 was activated at the shortest latency (*post-hoc* Wilcoxon signed-rank test; *z* = −2.79, *p* = 0.0052; *z* = −2.36, *p* = 0.0181; *z* = −3.33, *p* = 0.0009 for comparison to layers 2/3, 4, and 6, respectively) and layer 2/3 at longest latency relative to the others (*z* = −4.14, *p* = 0.00003; *z* = −2.80, *p* = 0.0051 for comparison to layers 4 and 6, respectively); layers 4 and 6 were not significantly different from each other. These data are consistent with those obtained from single cell experiments.

In comparing the laminar EPSP amplitude profile (Figure 3C) with the laminar spiking profile observed in electrophysiological recordings (Figures 5, 6), it is clear that this profile is not simply a function of the monosynaptic EPSP amplitude distribution. However, it is consistent with the laminar profile of resting potentials recorded in our whole-cell current clamp recordings (mean ± *SD*: E_L2/3_ = −78.1 ± 5.5 mV; E_L4_ = −70.1 ± 7.9 mV; E_L5_ = −65.2 ± 5.4 mV; E_L6_ = −72.3 ± 4.3 mV), which varied significantly by layer [One-Way ANOVA, *F*_(3, 93)_ = 24.96, *p* < 0.0000001]. Pairwise *post-hoc* tests (Tukey's HSD) showed that layer 5 cells had resting potentials that were significantly more depolarized than layer 2/3 cells (E_L2/3_-E_L5_ = −13.0 mV, 95% CI [−17.1 −8.9]), layer 4 cells (E_L4_-E_L5_ = −4.9 mV, 95% CI [−9.4 −0.44]), and layer 6 cells (E_L6_-E_L5_ = −7.1 mV, 95% CI [−12.1 −2.2]), similar to previous studies (Huggenberger et al., 2009; Constantinople and Bruno, 2013). In addition, spike thresholds did not vary by layer [One-Way ANOVA, *F*_(3, 93)_ = 0.31, *p* = 0.82], such that depolarized cells were closer to threshold.

Together, the data from our intracellular, on-cell and multiunit recordings provide compelling evidence that most spiking in auditory cortex in response to thalamic stimulation occurs during cortical network activity. Furthermore, this spiking does not appear to follow the spatiotemporal pattern predicted by the canonical microcircuit model. However, each of these electrophysiological techniques has associated limitations, and we used calcium imaging to corroborate these findings and explore the properties of these network events and the cells participating in them further. In particular, the calcium imaging technique allowed us to explore the spatial pattern of spiking probability as a function of cortical layer by simultaneously measuring spiking responses from large numbers of identified cells in layers 2–6.

### Calcium imaging technique

**Figure 7:**
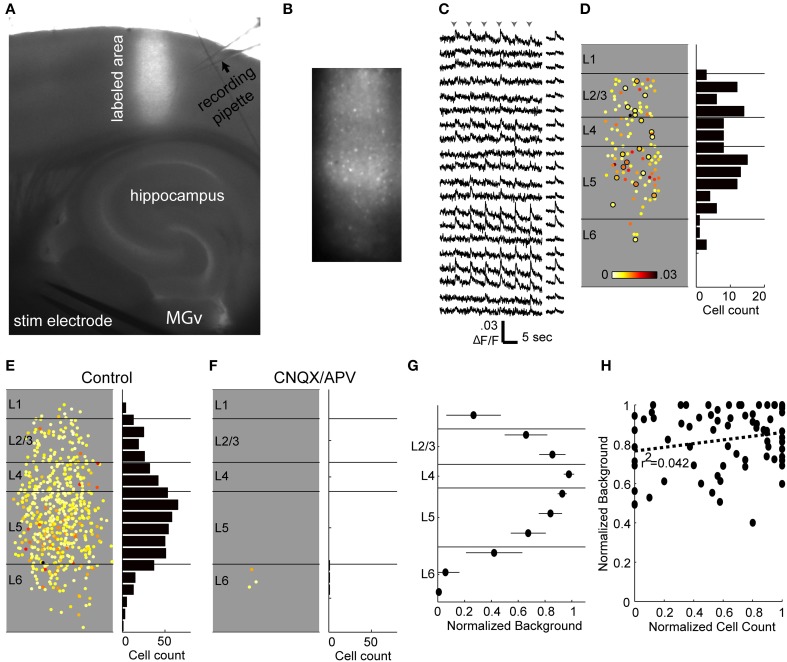
**Calcium imaging technique. (A)** Low-power combination fluorescence and brightfield micrograph shows OGB-1 dye loaded into a vertical strip of auditory cortex. **(B)** 10X response image taken by subtracting baseline fluorescence averaged over 1 s from 1 s of response to TC stimulation. Single responding cells are clearly seen as bright spots. **(C)** Sample fluorescence traces from a random selection of cells identified in the labeled region in **(B)** and sorted by depth (top, superficial; bottom, deep). Traces on the left are from single movies during which six stimuli (arrowheads) were presented 5 s apart and smoothed over 5 frames. Traces on the right are averages across stimulus presentations. Note that here and throughout, the units for fluorescence traces are ΔF/F, *not %Δ*F*/*F**. **(D)** *Left*, all responsive cells from the example experiment, plotted according to their location in the column and average change in fluorescence. Circled cells correspond to those with traces plotted in **(C)**. *Right*, spatial histogram of responding cell counts in 50 μm bins. **(E)** Cells responding to thalamocortical input in eight separate experiments, plotted on top of each other in normalized depth coordinates. **(F)** Responses from the same eight experiments in the presence of CNQX/APV. Almost all of the responding cells from **(E)** are blocked. **(G)** The background fluorescence signal was averaged within 100 μm depth bins and normalized to the brightest bin as a measure of labeling intensity. Points are plotted as mean ± standard deviation. **(H)** Normalized background as in **(G)** vs. cell count as in **(D)** (normalized within experiments to the layer containing the most spikes) for layers 2–5. Each point represents the background and cell count in a single 100 μm bin from a single experiment.

The calcium imaging technique employed here involves labeling a vertical strip of cortex with several injections of a membrane-permeable fluorescent calcium-sensing dye (OGB-1 AM; Figure 7A) and imaging at low enough power (10X) to capture most of the cortical depth (~80%) in a single field of view (Figures 7B–D). We did not image the most superficial 100 μm corresponding to layer 1, or the deepest 100–200 μm corresponding to deep layer 6. Consistent with these slices dwelling primarily in DOWN states, spontaneous spiking activity in cortical cells was rare (not shown). By contrast, afferent stimulation consistently elicited calcium transients in a subset of imaged cells (Figures 7B,C) presumably due to synaptically driven action potentials (Banks et al., 2011) (see below). The laminar profile of spiking activity was obtained by determining the position relative to the pia and white matter of each active cell (Figure 7D). Based on our electrophysiological recordings, we expected that most of these spikes occurred in the context of UP states, but we could not determine this directly because the time resolution of the calcium imaging (at least 30 ms due to the video frame rate and slower still due to the kinetics of OGB-1) is too slow to distinguish monosynaptically-driven spikes from those occurring during UP states. Instead, we recorded extracellularly in layer 5 to determine whether an UP state occurred on each trial, and used modified ACSF to reduce the likelihood of observing UP states on some trials (see below).

Cells in infragranular layers project to thalamus, and it is possible that electrical stimulation in these slices activated these cells antidromically and triggered the robust spiking activity observed in these layers. Antidromic activation is of particular concern because of previous reports *in vivo* that, at least under anesthesia, cortical network activity can be induced by brief activation of small numbers of layer 5 pyramidal cells (Stroh et al., 2013), or prolonged (3 min) activation of even single layer 2/3 pyramidal cells (Li et al., 2009). We investigated the incidence of antidromic activation in response to TC stimulation using the ionotropic glutamate receptor antagonists CNQX (10 μ M) and APV (40 μ M) to block excitatory synaptic transmission. Cells that continued spiking in the presence of CNQX/APV were assumed to be antidromically driven. In 8 experiments, only 3 out of 566 cells (0.5%) of cells that responded to TC stimulation spiked in the presence of CNQX/APV, and all were found in layer 6 (Figures 7E,F). We also did not encounter antidromically driven cells in any of the patch clamp experiments described above. The low percentage of cells insensitive to CNQX/APV suggests that antidromic activation, and network activity secondary to such activation, was minimal in our experiments.

Although ideally we sought to achieve uniform labeling across the ~800 μm of cortical depth that we targeted for imaging (i.e., layer 2—superficial layer 6) there was some variability in background labeling density, as measured by raw fluorescence as a function of cortical depth (Figure 7G). However, this variability in background did not have a strong effect on cell detection (Figure 7H). We compared background and cell count as a function of cortical depth by normalizing background and cell counts within experiments by their respective maxima and pooling these data across experiments. There was no significant correlation between background and cell count when layers 2/3, 4, and 5 were considered (Figure 7H; slope = 0.095, *r*^2^ = 0.04, *p* = 0.086). The correlation was significant if layer 6 was included, however the slope was small (slope = 0.22, *r*^2^ = 0.11, *p* = 0.0018) and therefore had minimal impact on cell counts; however we focused our analyses on layers 2/3 through 5.

**Figure 8:**
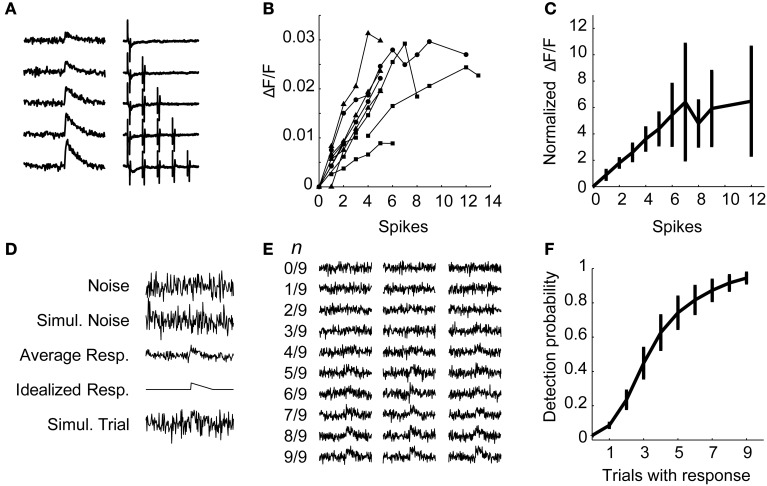
**Electrophysiological calibration of calcium signals. (A)** In an example calibration recording, averaged calcium traces (left) are sorted by the number of spikes simultaneously detected via on-cell recording (right). **(B)** Peak calcium transient (ΔF/F) plotted as a function of number of spikes detected using on-cell patch clamp for several cells. Each line represents a single cell, each data point represents the calcium change of the average of all traces in which that number of spikes was observed. Symbols indicate cells from different layers: layer 3 (*triangles*), layer 4 (*circles*), and layer 5 (*squares*). The change in calcium fluorescence is linear with spikes fired up to 6 spikes. **(C)** Data from **(B)** are normalized to the fluorescence change observed for the fewest spikes fired for that cell and averaged across cells. Data points are mean ± standard deviation. The normalized ΔF/F is linear up to about six spikes. **(D–F)** Simulated data was used to estimate the probability of detecting cells that fire less than one spike per trial. **(D)** The procedure for simulations is shown. For each calibration cell, the variance of the observed fluorescence noise on single trials was used to create Gaussian simulated noise. From the average one-spike response, an idealized response was derived by smoothing the data (5-point moving average) and measuring the peak. A single simulated trial is a simulated noise trial plus this idealized response. **(E)** Simulated averaged data consisted of 9 simulated trials, with *n* trials containing noise plus the idealized response and 9 − *n* trials containing only noise. Displayed fractions represent the number of trials containing the idealized response. Three examples of simulated averages are plotted for each trial composition. **(F)** Probability of detecting a response in the simulations as in **(E)** as a function of *n*, the number of trials containing a response. Data are plotted as mean ± s.e.m. across calibration cells.

Next, we verified that (a) we could measure the calcium transient due to single action potentials when averaging over a few trials, and (b) that the amplitude of the change in fluorescence is linear with the number of action potentials, by simultaneously recording optically and electrically from the same neurons. First, we delivered stimuli directly to cortex in the vicinity of an OGB-1 AM labeled area to evoke robust responses. Next, we obtained an on-cell recording from a randomly selected responding cell in layers 2–5 and simultaneously recorded fluorescence images at 10X and electrophysiological spiking responses to single pulses and brief trains of stimulation. Fluorescence traces on each trial were sorted according to the number of electrically identified spikes on that trial (Figure 8A). Plots of peak ΔF/F vs. number of spikes tended to be linear in individual cells but saturated in some cells at higher numbers of spikes (Figure 8B). After normalizing ΔF/F to the fewest spikes fired by a given cell and averaging these normalized values across cells, we find that the calcium-induced change in fluorescence in the population is linear with the number of action potentials fired up to about six spikes (Figure 8C). Cells that fired more than six spikes in response to a train of four stimuli varied in whether they continued to show near-linear increases in fluorescence with increases in spike count or whether they reached a plateau. Electrical stimuli delivered during the calibration experiments were strong, as they were delivered directly to cortex near the recording site, and we very rarely observed cells that fired more than six spikes in response to TC stimulation in patch clamp recordings.

Above, we presented evidence that cells in auditory cortex fire sparsely in response to stimulation of TC afferents, and thus most cells are unlikely to fire in response to every stimulus pulse in a train (Figures 5, 6A). To estimate our ability to detect cells that spike with a probability less than one, we simulated fluorescence data based on the calibration results (Figure 8D). Each simulation consisted of a mixture of *n* trials containing a simulated response (with a peak equal to the peak of the average one-spike calcium trace for that cell) embedded in Gaussian noise (generated with variance equal to the prestimulus variance for that cell), and 9 − n trials of Gaussian noise only, with *n* varied from 0 to 9 (Figure 8E). For each cell, this process was repeated 1000 times for each value of *n*. Using this procedure we detected a response in about 95% of the simulations that had a one-spike response on every trial of nine, and about 60–70% of simulations that had a one-spike response on four or five trials out of nine (Figure 8F). These experiments indicate that we can identify cells that spike consistently with a very high probability as well as a majority of those that fire with probability ~0.5 in response to single stimulation pulses. Note that in all of the calcium imaging experiments below, each trial consisted of trains of 4 pulses at 40 Hz. Thus, in these experiments, for a cell to fall below our detection limit 50% of the time, it would have to spike in response to less than 1 of 8 afferent stimulation pulses.

### Spatial pattern of spiking activity in response to TC stimulation

**Figure 9:**
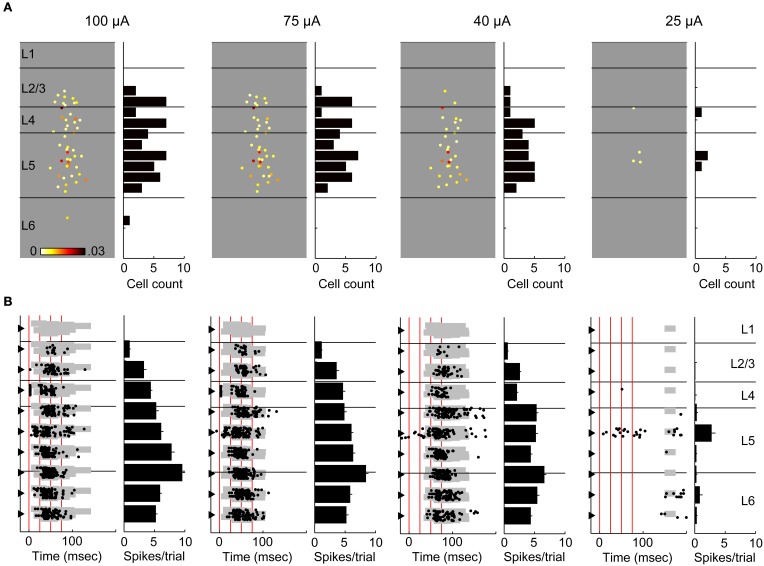
**Comparing calcium imaging with simultaneous multichannel recordings. (A)** An example experiment at four different stimulus intensities (100–25 μ A). At each intensity, responsive cells are plotted at their spatial position (*left*), color-coded to response amplitude. Color scale is 0–0.03 ΔF/F. Lines represent layer boundaries. To the right of each example experiment is a histogram count of the cells in 50 μm depth bins. **(B)** Simultaneous multichannel, multiunit recordings from the experiment in **(A)**. At each intensity, a spike raster (*left*) from 12 trials is plotted at the depth coordinate of each channel. The gray shaded area indicates detected UP states similar to Figure 5. Accompanying each raster plot is a depth histogram (*right*) showing the average number of spikes per trial on each channel. Error bars are s.e.m. Stimulus times are marked by vertical red lines. Note that early spiking activity on the electrode in layer 4 (visible immediately adjacent to the red line indicating stimulus time) is not present at lower stimulus intensities despite robust spiking associated with an evoked UP state. Also, for both techniques, spiking was very sparse at the lowest stimulus intensity (which did not reliably trigger UP states) and these sparse spikes included activity in layer 5.

We investigated the laminar spiking profile in response to activation of thalamic afferents using the calcium imaging technique. In a subset of experiments (*n* = 5 slices), we simultaneously monitored spiking activity by measuring MUA with multichannel electrodes (an example experiment shown in Figure 9). In this way, we could compare laminar spiking profiles obtained using the two techniques, both in the absence and presence of evoked UP states, and we could determine the contribution to the calcium responses of early spiking cells. At low stimulus intensities (Figure 9, *right column*), which either did not evoke UP states or evoked UP states with very long latency, spiking was limited to a small number of trials and a handful of cells located primarily in layers 4 and 5. Modest increases in stimulus intensity elicited UP states and dramatic changes in this spiking profile, with dense spiking observed throughout layers 2–6 (Figure 9, *Left three columns*). Consistent with the summary data shown in Figure 6D above, the MUA recordings in this example (Figure 9B) showed that the UP state spiking occurred earliest in layer 5 before spreading to other layers, with supragranular layers typically spiking at longest latency. Interestingly, in 4 of 5 slices, the threshold for early spiking appeared to be higher than the threshold for UP states, and short latency, well-timed spikes occurred only at the highest intensities and usually in layers 4 and 5 (Figure 9B). We also note that as stimulus intensity was increased, the spiking profile become relatively invariant, suggesting that above a certain threshold UP state activity saturated. In all cases, there was good correspondence between the laminar spiking profiles obtained using the imaging and electrophysiological techniques (Figure 9).

**Figure 10:**
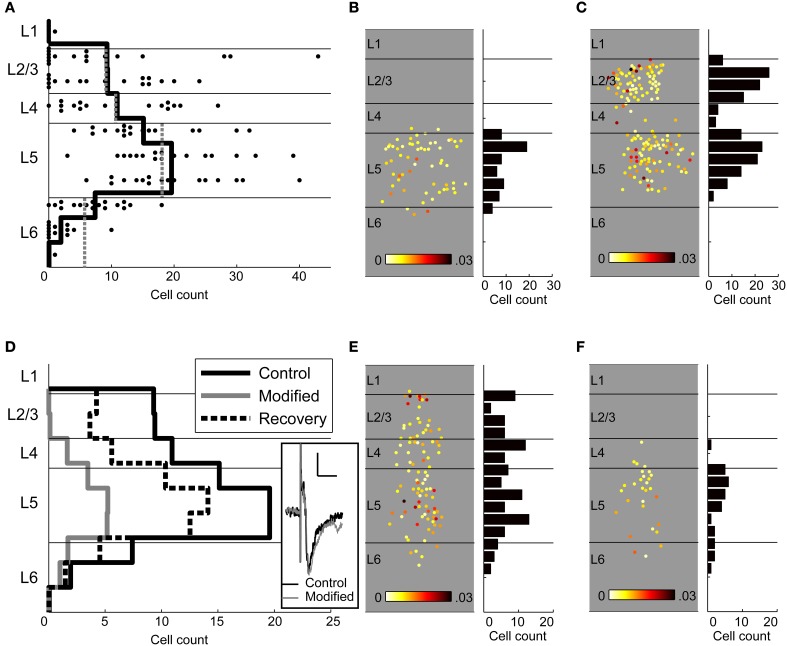
**Laminar profile of spiking responses. (A)** The number of responding cells detected using calcium imaging is plotted in 100 μm bins (*dots*) as well as the average number of cells per experiment (*solid black line*). In many experiments, there were few if any responding cells in layers 2/3, while every experiment showed robust activity in layer 5. The laminar distribution of cells observed spiking using calcium imaging in control conditions is nearly identical to the expected distribution based on on-cell recordings (*gray dotted lines*). **(B)** An example experiment in which thalamocortical stimulation primarily activated cells in layer 5, with no activity in layer 2/3. **(C)** In the same experiment, stimulation of a nearby cortical area triggered robust spiking in the imaged column, including in layers 2/3, demonstrating that the lack of layer 2/3 responses in **(B)** was not due to poor labeling or health of the superficial layers. **(D)** Cell counts in control conditions [*solid black lines*; same data as in **(A)**] show that layer 5 had a significantly greater density of spiking cells than the other layers. Modified ACSF (*solid gray lines;* modified ACSF contains elevated concentrations of divalent cations or 40 μ M APV) strongly reduced spiking across the cortical laminae, and layer 2/3 was more affected than the other layers. Modified ACSF eliminated UP state activity but did not affect the early component of the field potential response (*inset*; scale bar 10 ms, 0.2 mV). Recovery after high divalent cations (*thick dashed lines*) was similar to control cell counts. **(E)** An example experiment in which spiking cells were seen in all layers in normal ACSF. **(F)** The same experiment from **(E)** but with modified, high-divalent ACSF that blocks UP states. Far fewer cells are activated without UP states, and activity in layer 2/3 is completely blocked. Conventions for panels **(B,C,E,F)** are the same as the similar panels in Figures 7, 9.

We summarize the results of 17 calcium imaging experiments (including the 5 in which multichannel MUA was also recorded) in Figure 10. TC stimulation evoked spiking activity in layers 2 through 6, with peak density in layer 5 (Figure 10A, solid black line). We compared directly the observed pattern of spiking responses observed via calcium imaging to the pattern we observed in on-cell recordings, presented above in Figure 6 (see Materials and Methods). This spiking probability was strikingly similar to the cell count profile obtained using calcium imaging after normalizing to the cell count in layer 5 (Figure 10A, gray dotted lines). We performed a statistical analysis of cell counts from the calcium imaging data by fitting a generalized estimated equations (GEE) model using a Poisson distribution with logarithmic link function (see Materials and Methods). After normalizing for laminar thickness, layer 5 had a significantly greater density of responding cells than layers 2/3, 4, and 6 (exp(β_L2/3_-β_L5_) = 0.531, 95% CI [0.345 0.818], *p* = 0.004; exp(β_L4_-β_L5_) = 0.606, 95% CI [0.426 0.862], *p* = 0.005; exp(β_L6_-β_L5_) = 0.411, 95% CI [0.314 0.536], *p* < 0.000001). Other pairwise layer comparisons in control conditions were not significant.

We observed that robust activation of layer 5 was consistent across experiments, whereas activation in the granular and supragranular layers was more variable or perhaps bimodal (Figure 10A). In several experiments, activity was almost entirely absent in L2/3 during UP states recorded in layer 5. To ensure that layer 2/3 cells were healthy (able to spike in response to stimulation) in an experiment where spiking was almost entirely confined to layer 5 (Figure 10B), we also delivered electrical stimuli to cortex in layer 2/3 ~650 um from the recording site (Figure 10C). This stimulation triggered large UP states that included both layers 2/3 and 5, in contrast to thalamic stimulation. These data suggest that the involvement of supragranular layers in UP states was variable, but not because of the health of the tissue or inadequate labeling. We will return to this issue below.

In those experiments in which we wished to isolate early spike responses from spiking associated with UP states, we suppressed polysynaptic activity and blocked UP states using either high divalent cations ([Ca^2+^] = 4.0 mM, [Mg^2+^] = 4.2 mM; *n* = 13 experiments), which raise spike thresholds and preferentially block polysynaptic activity, or the NMDA receptor antagonist APV (40 μM) (Berry and Pentreath, 1976; Metherate and Cruikshank, 1999; Cruikshank et al., 2002; Rose and Metherate, 2005) (Figure 10D). These procedures left monosynaptic TC synaptic responses relatively unaffected (Figure 10D, *inset*). We note that a limitation of this method is that the modified ACSF will also raise spike thresholds in cortical cells and thus decrease even monosynaptic spike probability, and that this effect may be cell type-specific. If this is the case then some cell types recorded in modified ACSF may be overrepresented in our sample. However, 290/312 cells observed spiking in the absence of UP states in modified ACSF also spiked in control conditions, indicating that cells observed in modified ACSF were rarely made *more* likely to fire by the modified ACSF, and thus nearly all these cells participated in early spiking activity under control conditions as well. For our experiments it was important to use methods that selectively block evoked UP state activity rather than silencing the cortex, e.g., with muscimol, because we are looking for superthreshold responses that would be blocked by cortical silencing. Below, first we characterize how modified ACSF altered the laminar spiking profile, and then describe the properties of cells targeted for patch clamping under modified vs. control ACSF.

TC stimulation in ACSF modified by high divalent cations or APV resulted in less spiking activity than control conditions (exp(β_hDiv/APV_) = 0.102, 95% CI [0.056 0.185], *p* < 0.000001), as expected due to the suppression of UP states (Figure 10D, gray line). Although modified ACSF resulted in fewer responding cells in every layer, this effect was not uniform across layers, as is clear in an example experiment showing responding cells in control (Figure 10E) and modified (high divalent cations, Figure 10F) ACSF. The interaction between layer and modified ACSF was significant [Wald χ^2^_(3)_ = 33.017, *p* = 0.00000032]. Pairwise interaction comparisons were significant between layers 2/3 and 4 (exp(β_L2/3^*^ACSF_−β_L4^*^ACSF_) = 0.082, 95% CI [0.026 0.256], *p* = 0.000018), layers 2/3 and 5 (exp(β_L2/3^*^ACSF_−β_L5^*^ACSF_) = 0.048, 95% CI [0.015 0.158], *p* = 0.0000005), and between layers 2/3 and 6 (exp(β_L2/3^*^ACSF_−β_L6^*^ACSF_) = 0.053, 95% CI [0.014 0.203], *p* = 0.000018). These interactions indicate that layer 2/3 is more affected by suppression of UP states than are the other layers. Other pairwise comparisons were not significant, but layer 5 was the least affected by suppression of UP states. If layer 5 is primarily driven by layer 2/3, as suggested by the canonical microcircuit, blocking polysynaptic activity would be expected to affect layer 5 more strongly than layer 2/3. These results instead suggest that layer 5 is strongly activated by TC input, independently of the activity of the supragranular and granular layers, as recently shown *in vivo* (Constantinople and Bruno, 2013).

To confirm that cells spiking in modified ACSF were in fact being driven by monosynaptic TC EPSPs, and to identify these early spiking cells' morphology and electrophysiology, we identified specific cells spiking in response to TC stimulation via their fluorescence signals and targeted them for patch clamp recording and subsequent classification. The protocol for these targeted patch experiments was to bathe the slice in modified ACSF, verify the absence of UP states, identify a cell still spiking in the absence of UP states, and obtain a whole-cell recording from that cell. All six cells in layer 5 that were identified via their fluorescence signals as spiking in modified ACSF in the absence of UP states had spikes that were monosynaptically driven by TC stimulation. Three of these cells spiked in response to a single TC stimulus, while the other three spiked in response to stimuli two through four of a 40 Hz train of TC stimuli. Interestingly, none of these targeted, early spiking cells were pyramidal cells. Five cells had morphology and spiking patterns consistent with Martinotti interneurons (Kawaguchi and Kubota, 1996, 1997, 1998; Markram et al., 2004; Wang et al., 2004). The sixth cell had a non-pyramidal, non-spiny, bipolar morphology and a stuttering spiking pattern.

By contrast, we also targeted 7 cells identified via their fluorescence signals as spiking in normal ACSF under control conditions, i.e., in the presence of UP states. All 7 cells were identified as pyramidal cells based on their morphology and firing properties (either regular spiking or burst firing). Only one of these cells fired short latency, well-timed spikes prior to the onset of UP states; the other six cells fired spikes only during UP states. Including cells targeted in modified and normal ACSF and our random whole-cell recordings presented above (Figure 3D), 9 out of 11 cells that we observed spiking in direct response to monosynaptic EPSPs, outside the context of UP states, were putative interneurons, and 7 of these cells plus two pyramidal cells were located in layer 5. This result is even more striking given the low percentage of cells in neocortex that are GABAergic interneurons (~20%; Markram et al., 2004; Ascoli et al., 2008). The cells targeted in the absence of UP states were significantly more likely to be non-pyramidal cells than those targeted in the presence of UP states (Fisher's exact test, *p* = 0.0047), indicating that there was not simply a greater likelihood of choosing non-pyramidal cells via targeted patching.

### Variable activation of layer 2/3 during UP states

**Figure 11:**
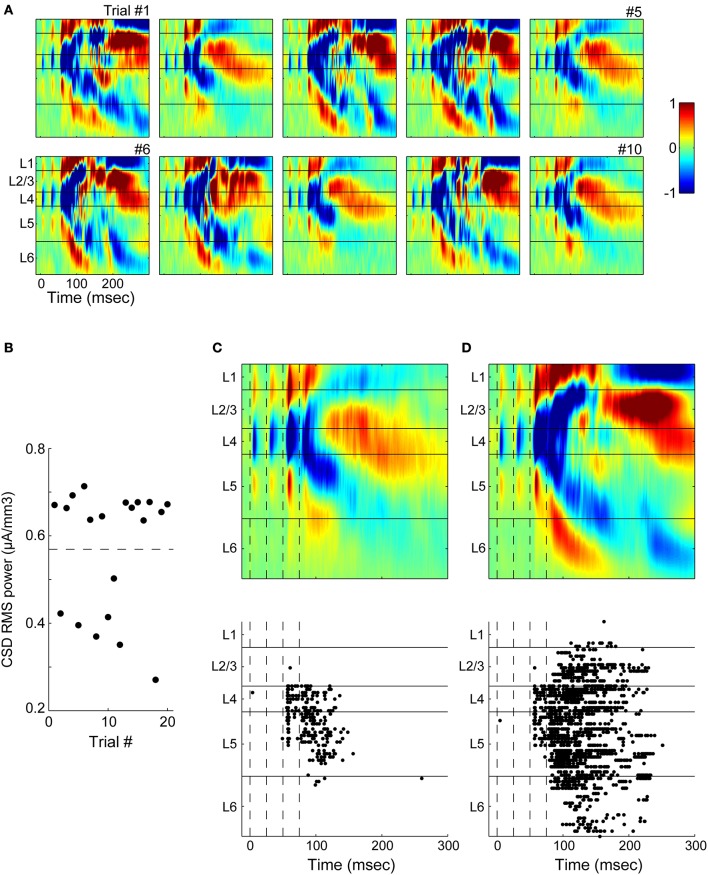
**Variable supragranular involvement in UP states in CSD and MUA recordings. (A)** Observed UP states varied bimodally in intensity. Each panel is the CSD of a consecutive single trial, and all trials were at a constant stimulus intensity. Note two classes of responses differing in intensity (e.g., Trial #1 compared to Trial #2). **(B)** Twenty consecutive trials, including those in **(A)**, were separated into small and large UP states based on the root-mean-squared CSD power across channels. Each point represents a single trial, and the horizontal line separates the bimodal distribution of responses. **(C,D)** CSD responses (*top*) averaged over the small **(C)** and large **(D)** UP states from **(B)**. Multiunit activity (*bottom*) from these same sets of trials was measured on the same electrode array used for CSD recording. Spike data are plotted in raster form, one line for each trial, with trials from different channels separated by a vertical space. On trials for which the CSD showed weak UP states, multiunit spiking activity was observed in layers 4 and 5 but very little in layers 2/3. Trials that showed strong UP states in the CSD showed more multiunit activity in all layers but also involved layer 2/3. Color scale is ±1 μA/mm^3^ for **(A,C,D)**.

**Figure 12:**
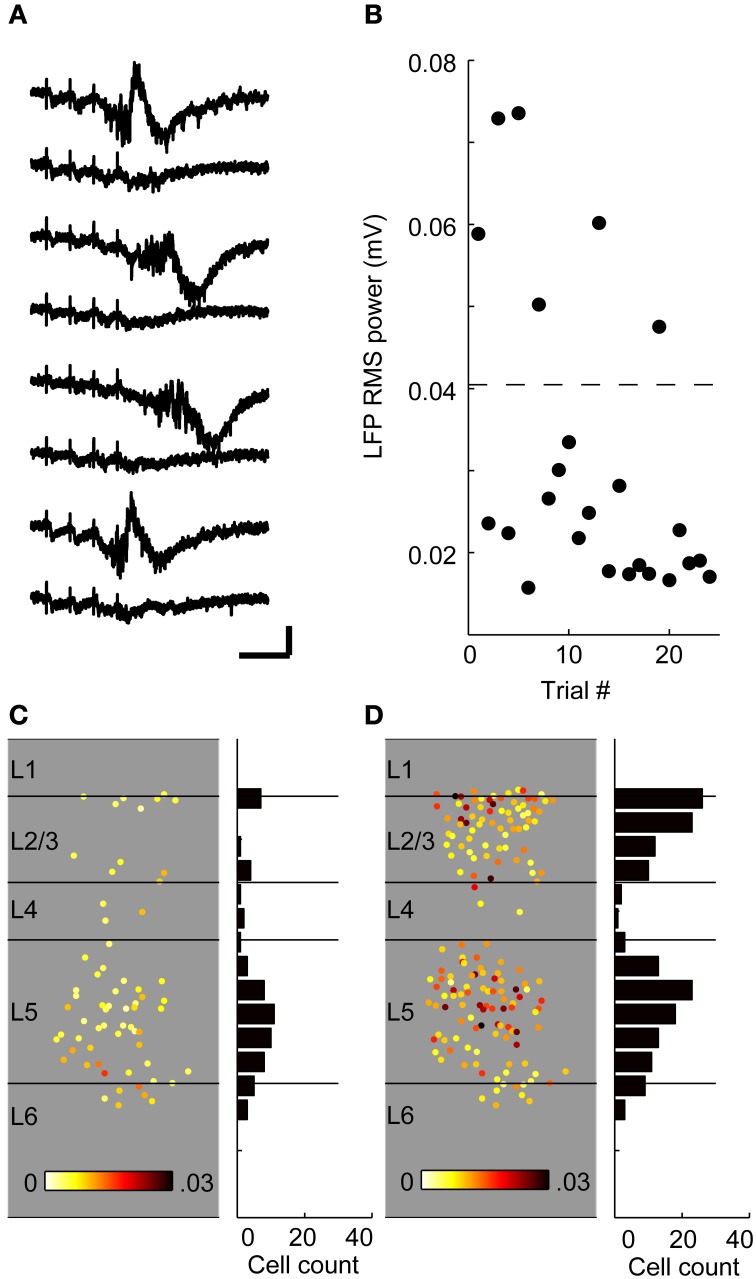
**Two classes of UP states in calcium imaging. (A)** Shown are LFPs recorded in layer 5 during consecutive trials of a single calcium imaging experiment demonstrating two sizes of UP states (using the same stimulus intensity on all trials). Scale bar: 50 ms, 0.1 mV. **(B)** Calcium traces were sorted according to the power of the LFP, similarly to the experiment in Figure 11. Six trials had large UP states, so data from only 6 of the 18 trials (randomly chosen) with small UP states were analyzed. For trials with small UP states **(C)**, most of the responsive cells were found in the infragranular layers. Trials with large UP states **(D)**, were associated with much more robust calcium responses in all layers, with an especially striking change in the superficial layers. Color scale for **(C,D)** is 0–0.03 ΔF/F.

The data of Figure 10 suggest that across experiments, the involvement of supragranular layers in UP states is more variable than that of infragranular layers: whereas layer 5 participated strongly in all experiments, layer 2/3 involvement was less consistent. This variability was also manifested on a trial-by-trial basis, even when the stimulus intensity was the same on each trial, both in extracellular electrophysiology (Figure 11) and calcium imaging (Figure 12) experiments. In extracellular recordings, we observed two types of UP states in CSDs that differed in their intensity, duration and in their involvement of supragranular layers (Figure 11A). In the example illustrated, TC responses in granular layers to a train of four stimuli on trials 1, 3, 4, 6, 7, and 9 first spread to layer 5 and then to supragranular layers as well as layer 6 over the next 200 ms (Figure 1A). By contrast, activity spread to layer 5 and triggered a modest current sink in layer 2 before dying out on trials 2, 5, 8, and 10. These visually distinct responses could also be separated on the basis of the total CSD power during the response window 0 < t < 300 ms (Figure 11B). Averaged CSDs of these two types of responses (Figures 11C,D, *top*) showed far larger sinks in supragranular layers for the larger UP states than for the smaller, and simultaneously measured MUA (Figures 11B,C, *bottom*) showed little involvement of layer 2/3 in superthreshold activity on trials with smaller UP states but robust activation on trials with larger UP states. In other CSD experiments (not shown), small increases in stimulus intensity biased the network toward the larger UP states, indicating that involvement of all layers could depend on stimulation being of sufficient strength.

Distinct UP states sizes and associated laminar response patterns were also observed in calcium imaging experiments (Figure 12). In the example illustrated, trials were sorted according to two clearly distinguishable response classes in the local field potential recorded in layer 5 (Figures 12A,B). Calcium traces in individual cells were larger and more cells were active, especially in the superficial layers, on the trials with a “larger” UP state (Figures 6C,D).

## Discussion

### Thalamically-evoked activity patterns in auditory cortex

**Figure 13:**
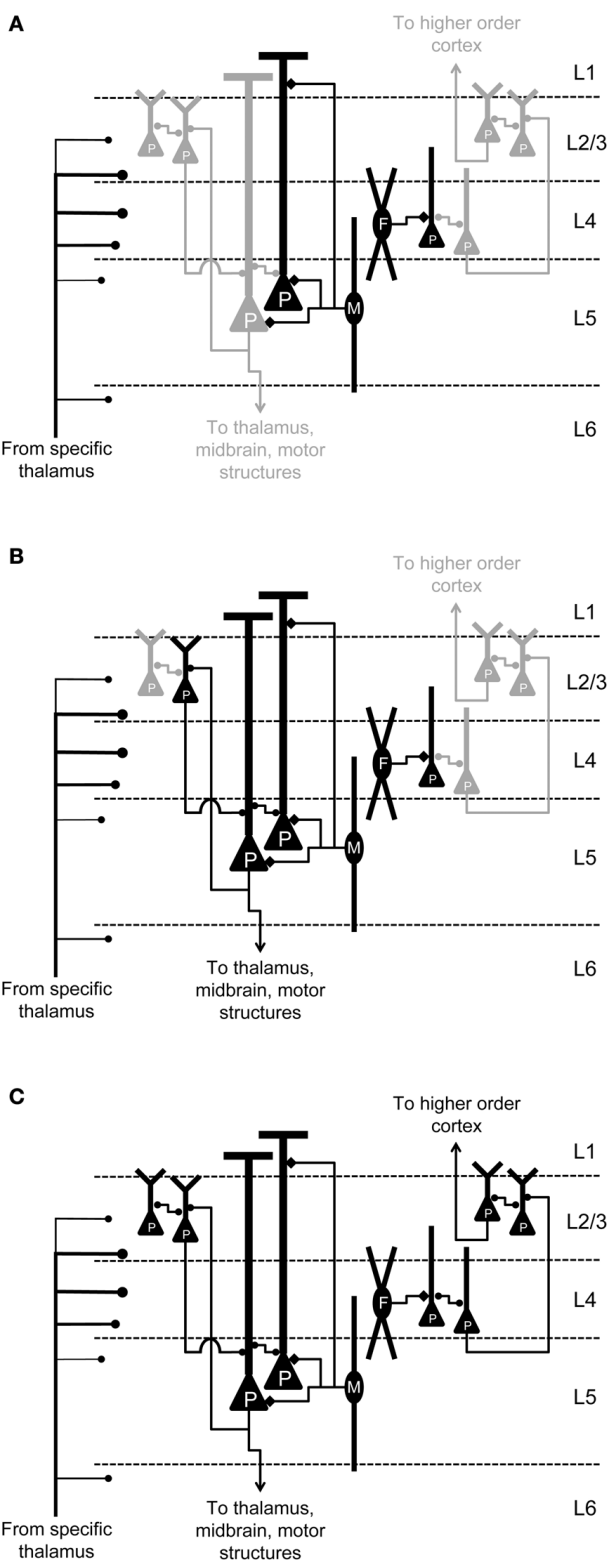
**Diversity of activity patterns in response to thalamic stimulation**. Circuit diagrams of auditory cortex summarizing key findings from this study and previous reports. Pyramidal cells (triangles) and GABAergic interneurons (ovals; M, Martinotti cells; F, fast-spiking cells) are depicted along with some of their local connections. Active cells are in black, inactive cells are in gray. **(A)** Thalamic stimulation elicits monosynaptic responses primarily in GABAergic cells and a small number of pyramidal cells in layers 4 and 5. **(B)** For stimuli of sufficient intensity and after a delay of ~10–100 ms, UP state activity emerges in the infragranular network. **(C)** Strong stimuli trigger UP state activity that spreads to supragranular layers as well.

We distinguished between two types of spiking responses to thalamic stimulation. In murine auditory cortex, most cells that spike do so during brief network bursts that occurred nearly simultaneously in cells throughout the cortical region; these cells depolarize in response to a barrage of polysynaptic activity and fire occasional spikes during these events. By contrast, only a small number of cells fire action potentials triggered at short latency by monosynaptic TC EPSPs (Figure 3D), even though short latency, monosynaptic, subthreshold synaptic responses are observed in cells throughout layers 2–6. We summarize our results in the circuit diagrams of Figure 13. In this model, monosynaptically driven spiking occurs predominantly in (putative) GABAergic interneurons, including Martinotti and fast spiking cells (Figure 4), as well as an occasional pyramidal cell. Stimuli of sufficient intensity elicit UP states involving robust spiking activity (Figures 13B,C). UP state activity occurs primarily in pyramidal cells, and appears with earliest latency in pyramidal cells in layer 5, consistent with previous reports (Chauvette et al., 2010; Wester and Contreras, 2012; Beltramo et al., 2013). In some cases, UP state activity is largely confined to infragranular layers (Figure 13B), where dense interconnectivity between pyramidal cells and depolarized resting potentials allow for sustained network events lasting ~100 ms. Functionally, ascending information during these smaller UP states flows back to subcortical structures and may engage cortico-TC loops, but does not engage direct cortico-cortical hierarchical processing. In other cases, likely only in response to stimuli of sufficient intensity or simultaneous ascending and descending input, UP state activity spreads throughout the column and involves supragranular layers as well (Figure 13C), potentially activating direct cortico-cortical pathways.

### Dense spiking in infragranular layers

We observed that short latency and robust spiking in response to thalamic stimulation occurred in layer 5 cells during evoked UP states (Figures 6, 10D), and that even in the absence of UP states layer 5 cells could still dominate the spiking responses to TC stimulation. This stands in contrast to the predictions of the canonical microcircuit model, in which the shortest latencies to thalamic stimulation occur in layer 4 spiny stellate cells. Indeed, we would have expected that in the absence of polysynaptic activity (i.e., at low stimulus intensities or in modified ACSF), spiking would have been dominated by cells in layer 4, rather than cells in layer 5, as we observed (Figures 9, 10). However, spiny stellate cells are rare in auditory cortex (Smith and Populin, 2001; Barbour and Callaway, 2008; Sakata and Harris, 2009), and here and elsewhere in cortex pyramidal cells in layers 2–6 with dendrites in thalamo-recipient layers are likely excited directly (Figures 3A–C). These results suggest that layer 5 cells get more direct thalamic input than is appreciated based on afferent terminal density (Gil and Amitai, 1996; Bureau et al., 2006; De Kock et al., 2007). Additionally, cells in layer 5 have more depolarized resting potentials and lower spike thresholds than cells in other layers (see Results) similar to previous results *in vitro* (Huggenberger et al., 2009) and *in vivo* (Manns et al., 2004; Constantinople and Bruno, 2013). A recent study in somatosensory cortex demonstrated direct and early activation of layer 5 cells, independent of granular layer activation (Constantinople and Bruno, 2013). Consistent with these observations, thick-tufted pyramidal cells *in vivo* are the earliest and most likely to be activated by whisker deflection in barrel cortex (De Kock et al., 2007) and most likely to be activated by auditory tones or clicks in auditory cortex (Sakata and Harris, 2009). The purpose of a highly excitable recurrent layer 5 network may be to trigger arousal in response to strong stimuli (Harris and Thiele, 2011), or perhaps to evoke “packets” of information-carrying activity (Luczak et al., 2013), which have a similar duration to the evoked UP states we observed. If layer 5 cells are both highly responsive to input and directly activated by thalamus, connections from layer 5 to other layers (particularly supragranular layers) may contribute a greater fraction of synaptic current than the number of collateral fibers or response to focal stimulation would suggest. Thus, activation of layer 5 cells by TC input may provide a pathway through which information can ascend the cortical hierarchy, either through UP states and subsequent activation of supragranular cells, or through a cortico-TC circuit (Guillery and Sherman, 2002; Theyel et al., 2010) without active processing in supragranular layers (Constantinople and Bruno, 2013).

### Monosynaptic TC activation of non-pyramidal cells

We observed that cells that spiked at short latency to TC stimulation, in the absence of UP states, were most often non-pyramidal, and had firing patterns and morphology consistent with either fast spiking parvalbumin-positive cells (Kawaguchi and Kubota, 1993) (in layers 4 and 5) or low-threshold/burst-spiking somatostatin-positive Martinotti cells (in layer 5) (Kawaguchi, 1993; Kawaguchi and Kubota, 1997; Wang et al., 2004). The spikes from these latter cells sometimes occurred with a considerable delay after the stimulus (up to 15 ms) and appeared to result from a slow, intrinsic conductance triggered by the initial depolarization similar to that seen with just-threshold current injection.

Preferential activation of fast spiking interneurons by TC afferents has been described previously in somatosensory (Beierlein et al., 2003; Gabernet et al., 2005; Cruikshank et al., 2007) and auditory cortex (Schiff and Reyes, 2012), where these cells are postulated to mediate feedforward inhibition that restricts integration windows and sharpens spike timing. Evidence for short-latency activation of infragranular Martinotti cells is mixed. Several studies have found that these cells, whose axons synapse on the dendrites and apical tufts of pyramidal cells in layers 1, 4, and 5 (Markram et al., 2004; Wang et al., 2004), are only weakly excited by TC afferents compared to fast-spiking interneurons (Gibson et al., 1999; Verbny et al., 2006; Cruikshank et al., 2010). In contrast, others have shown that somatostatin-positive Martinotti cells in layers 2/3 are closer to threshold than regular- and fast-spiking cells at rest (Fanselow et al., 2008) and that layer 5 somatostatin-positive interneurons can mediate feed-forward inhibition (Tan et al., 2008); however, the population sampled in the latter work likely did not include the Martinotti cells specifically (see Ma et al., 2006). Our results support the notion that both fast spiking and Martinotti cells are directly involved in, and may even dominate, the monosynaptic cortical response to thalamic input. Because many of the early-activated interneurons are located in layer 5, where evoked UP states are likely to originate (Sanchez-Vives and McCormick, 2000; McCormick et al., 2003; Sakata and Harris, 2009; Chauvette et al., 2010; Wester and Contreras, 2012; Beltramo et al., 2013), these cells may be involved in regulating the onset of evoked UP states.

Although we did not systematically test for the presence of disynaptic (i.e., feedforward) inhibition in every pyramidal cell recorded, of the 33 cells in which we did investigate this we observed short latency inhibitory responses in only 8 cells. This observation is consistent with previous reports in auditory cortex (Hefti and Smith, 2003; Rose and Metherate, 2005; Verbny et al., 2006). A heavy contribution by Martinotti cells to feedforward inhibition in auditory cortex could explain why disynaptic inhibition is not observed more commonly. Because these cells are dendrite-targeting interneurons (Kawaguchi and Kubota, 1997; Markram et al., 2004), these electrotonically-remote inhibitory conductances are unlikely to be well-observed at the soma. This may explain the low percentage of cells with observed disynaptic inhibition relative to the likelihood of putative interneurons to spike in our recordings.

### Variable activation of supragranular layers during evoked UP states

Whereas cells in layer 5 were consistently activated during UP states, activation of cells in layer 2/3 was more variable (Figures 10–12). The paucity of spiking observed in supragranular layers is consistent with previous reports of sparse spiking in layer 2/3 (Barth and Poulet, 2012), and may be a feature of how these cells encode sensory information. It is also possible that once the supragranular network is activated, spiking in layer 2/3 cells is as dense as in layer 5. UP states that involved layer 2/3 were more intense than those that were confined to infragranular layers, and spiking during these larger UP states in supragranular layers was as dense as in infragranular layers (Figures 11, 12). In some experiments, we observed that the size of the UP state and the involvement of layer 2/3 depended on stimulus intensity (not shown), suggesting a model in which the cortical microcircuit gates ascending sensory input: only selected inputs activate layer 2/3 and incorporate into direct cortico-cortical hierarchical processing, whereas “packets” (Luczak et al., 2013) of spikes occur readily in infragranular layers to provide rapid motor output, and possibly activate cortico-TC loops. This differential involvement of supragranular layers has been observed before in auditory cortex *in vivo*, where spontaneous UP states were shown to only on occasion spread from infra- to supragranular layers (Sakata and Harris, 2009). Probabilistic involvement of layer 2/3 in these network events is consistent with recent observations that layer 2/3 activity occurs as population bursts whose probability depends on stimulus parameters in a non-linear fashion (Bathellier et al., 2012).

The observation of differential involvement of layers 5 and layer 2/3 in UP states adds to a growing body of evidence that activity in infra- and supragranular layers can be uncoupled and may be linked to different cortical processes. For example, activity in infra- and supragranular layers exhibit different spectral characteristics (Maier et al., 2010), and activity in supragranular layers has been more closely linked to attention and perceptual awareness (He and Raichle, 2009; Buffalo et al., 2011). “Avalanches” in cortical networks have also been observed exclusively in supragranular and not infragranular networks (Beggs and Plenz, 2003; Gireesh and Plenz, 2008; Petermann et al., 2009). These observations are also clearly incompatible with the prediction of the canonical microcircuit that activity in infragranular layers is driven by activity in supragranular layers, and support instead a model of two parallel, interacting networks, as proposed in the original formulation of the model (Douglas et al., 1989; Douglas and Martin, 1991).

### Concurrence of electrophysiological and calcium imaging data

Although our calcium imaging technique is less sensitive than those employing two-photon microscopy, our simultaneous on-cell/calcium imaging calibration experiments (Figure 8) demonstrate that we are able to detect single spikes in cells from all layers. Our main findings using calcium imaging, i.e., that spiking is densest in layer 5 following TC stimulation, that early spiking cells tend to cluster in layers 4 and 5 and are mostly non-pyramidal, and that the participation of layer 2/3 in UP states is variable, were all corroborated with parallel electrophysiological recordings. In addition, we reported calcium responses as the number of responding cells rather than the amplitude of the response in part to avoid bias toward cell populations that are larger, fire bursts of spikes or have lower concentrations of endogenous calcium binding proteins. Thus, the laminar profile of spiking probability following TC stimulation obtained with calcium imaging was nearly identical to that obtained with random on-cell patch experiments (Figure 10A). In addition, the cells recorded using targeted patch experiments in modified ACSF were mostly interneurons, similar to the cells recorded using random whole-cell patch recordings (Figure 4). We note that interneurons such as those targeted are smaller than pyramidal cells and likely have high concentrations of calcium buffers (Lee et al., 2000; Aponte et al., 2008), which if anything should make them harder to detect in calcium imaging experiments than pyramidal cells. Although these cells do fire bursts of action potentials, which could make them easier to detect despite their small size, the cells targeted in modified ACSF were not exclusively burst-firing cells, and our calibration experiments showed that cells that fired one spike per trial were easily detected (Figure 8).We also noted (Figure 8C) that there is a reduced probability of detecting cells that spike less than once per trial (i.e., <1 spike per four afferent stimulus pulses). Because layer 2/3 cells are reported to fire more sparsely *in vivo* than layer 5 cells (Sakata and Harris, 2009, 2012), we were concerned that these detection limits in our calcium imaging technique may underestimate the participation of L2/3 cells in activity patterns generated by TC stimulation. However, spiking probability in L2/3 estimated via on-cell recordings was consistent with that observed during calcium imaging, though cells in supragranular layers were still observed to fire more sparsely, and at longer latency, than infragranular or granular cells. The variable involvement of these layer 2/3 cells in evoked UP states observed in calcium imaging experiments was also observed in multichannel electrode recordings (Figures 11C,D).

### Functional implications

The data and model presented here present have far-reaching implications for understanding the state-dependence of information processing in auditory cortex. In particular, the observation that supragranular layers may only participate in UP state activity under certain conditions may be relevant to top-down, state-dependent modulation of cortical sensory responses. The low efficacy with which thalamic afferents drive monosynaptic spiking activity in cortex has been noted previously (MacLean et al., 2005), and recent reports emphasize the importance of cortical network activity in responses to sensory stimuli *in vivo* (Bathellier et al., 2012; Hromadka et al., 2013; Luczak et al., 2013). It is clear that extrapolating from the observations presented here to functional interpretations *in vivo* is challenging due to the limitations of the brain slice preparation. In our experiments, the absence of intact cortical networks and the resulting relative quiescence network state at rest, the limited influence of subcortical neuromodulators, and the substitution of synchronous fiber bundle stimulation for sensory input all constrain the range of possible cellular and network responses to a subset of those available *in vivo*. In spite of these limitations, however, there are striking similarities between the responses observed here and those observed *in vivo*. For example, UP states in our slices had durations that ranged from ~50 to 200 ms, similar to those reported *in vivo* for UP states (Sakata and Harris, 2009) and “bumps” (Hromadka et al., 2013) after accounting for temperature differences between preparations. The variable involvement of supragranular layers in UP states that we observed is similar to that reported for spontaneous UP states *in vivo* (Sakata and Harris, 2009), as is the observation of early activation of layer 5 cells (Constantinople and Bruno, 2013). Thus, we suggest that the network activity elicited by repetitive stimulation of TC afferents under control conditions in our slices is similar to the UP state-associated activity elicited by sensory stimulation *in vivo* (Sakata and Harris, 2009; Luczak et al., 2013). However, in the intact cortico-thalamic network, other afferent inputs and neuromodulators likely bias the network state toward or away from UP state activity and may particularly influence the involvement of the superficial layers.

The stochastic nature of UP states evoked by TC stimulation, for example the variability of their onset latency and duration on successive trials (Figure 5), as well as their characterization as intracortically-mediated events, raise the question as to whether spikes occurring during UP states are time-locked to the TC stimuli and can carry information about specific temporal features of the TC input train. The observation that most spiking occurs during these intracortically-mediated events is particularly surprising in auditory cortex, given the importance of spike timing in the ascending auditory pathway for sensory coding. Although we observed some evidence for consistent spike timing across trials in the context of UP states [e.g., in the raster plot for the deepest layer 5 cell (Figure 5B), in which spikes occurring between *t* = 25 and 50 ms on each trial tend to line up vertically, indicating consistent timing on successive trials], in other cells this was not the case. Further experiments aimed specifically at information coding will be better able to quantify spike jitter during UP states and the implications for encoding and decoding.

### Conflict of interest statement

The authors declare that the research was conducted in the absence of any commercial or financial relationships that could be construed as a potential conflict of interest.
